# Are differences in travel time or distance to healthcare for adults in global north countries associated with an impact on health outcomes? A systematic review

**DOI:** 10.1136/bmjopen-2016-013059

**Published:** 2016-11-24

**Authors:** Charlotte Kelly, Claire Hulme, Tracey Farragher, Graham Clarke

**Affiliations:** 1 Leeds Institute of Health Sciences, University of Leeds, Leeds, UK; 2 Institute for Transport Studies, University of Leeds, Leeds, UK; 3 School of Geography, University of Leeds, Leeds, UK

**Keywords:** Systematic Review, Access to Healthcare, Health Outcomes

## Abstract

**Objectives:**

To investigate whether there is an association between differences in travel time/travel distance to healthcare services and patients' health outcomes and assimilate the methodologies used to measure this.

**Design:**

Systematic Review. We searched MEDLINE, Embase, Web of Science, Transport database, HMIC and EBM Reviews for studies up to 7 September 2016. Studies were excluded that included children (including maternity), emergency medical travel or countries classed as being in the global south.

**Settings:**

A wide range of settings within primary and secondary care (these were not restricted in the search).

**Results:**

108 studies met the inclusion criteria. The results were mixed. 77% of the included studies identified evidence of a distance decay association, whereby patients living further away from healthcare facilities they needed to attend had worse health outcomes (eg, survival rates, length of stay in hospital and non-attendance at follow-up) than those who lived closer. 6 of the studies identified the reverse (a distance bias effect) whereby patients living at a greater distance had better health outcomes. The remaining 19 studies found no relationship. There was a large variation in the data available to the studies on the patients' geographical locations and the healthcare facilities attended, and the methods used to calculate travel times and distances were not consistent across studies.

**Conclusions:**

The review observed that a relationship between travelling further and having worse health outcomes cannot be ruled out and should be considered within the healthcare services location debate.

**Strengths and limitations of this study**

- This systematic review has, for the first time, synthesised available evidence on the association between differences in travel time/distance to healthcare services and patients' health outcomes.
- It has identified a wealth of studies and generated evidence for a wide range of disease groups and health outcomes, across multiple countries.
- The review found great variation in study design, distances and travel time to access healthcare settings, and range of health outcomes; this precluded pooling of data for a meta-analysis.
- While the review findings are of undoubted value in broadening our understanding of the wider societal factors that influence health outcomes, their applicability may be limited to countries with similar healthcare systems.

## Introduction

Countries, such as the UK, USA and Canada, have been implementing a policy of centralising the care of patients for many specialised services. There is evidence that this process will have a positive impact on the health outcomes of those patients treated in these specialised centres.^1^ ^2^ However, there are also drawbacks to increasing the distance some patients travel to receive treatment. A number of authors have documented the *distance decay* association, which identifies that those who live closer to healthcare facilities have higher rates of usage after adjustment for need than those who live further away.^3^ ^4^ Indeed, as long ago as 1850 Jarvis proposed this distance decay effect by finding that fewer patients were admitted to a psychiatric hospital in Massachusetts the further they lived from that hospital.^5^ While there is evidence of this distance decay association, there is less evidence on how this translates into impacts on health outcomes. Having to travel further to access healthcare facilities and the impact this has on patients health require further investigation.

A growing number of studies have determined transport accessibility levels to healthcare using geographical information system (GIS) techniques, by mapping car and public transport travel times and distances to healthcare facilities. These can be broadly split into *revealed accessibility and potential accessibility methods, as defined by Khan.*^6^ Revealed accessibility refers to methods that use data from actual healthcare trips, for example, the drive time or straight-line distance between a patient's home address and the hospital they attended.^7^ ^8^ Potential accessibility refers to methods that look at what is the potential for accessing healthcare facilities in a particular area, for example, using gravity models^9^ and specialised gravity models—such as, two-step flotation catchment area method.^10^ ^11^ While these methods are being widely used and developed, the link between transport accessibility to healthcare and the association of this with patients' health outcomes has not frequently been considered (in part due to a lack of linked health and transport accessibility data). The aim of this review is to bring together studies that have calculated accessibility (patients travel to healthcare facilities—ex-post) and explored whether there is an associated impact from this on health outcomes. The focus lies on whether there is an association and the data and methods used.

## Methodology

The review protocol was published in advance on the PROSPERO database (CRD42014015162). The study followed the PICOS (Population, Intervention, Comparator, Outcome, Study type) search design.^12^ The population were adults accessing healthcare in global north countries (studies were included from the following regions/countries: Northern America, Western Europe, Australia and New Zealand). The intervention and comparator were the distance and travel times to healthcare. The outcomes were any health outcomes (eg, survival, mortality and quality of life) and proxy measures for health outcomes (eg, follow-up attendance and usage of clinics). No restriction was made on study type or design. We searched Web of Science, MEDLINE, Embase, Transport database, HMIC and EBM Reviews for relevant papers in November 2014 and updated the search on 7 September 2016. The MEDLINE search strategy is accessible in online supplementary material 1. All titles and abstracts were screened by CK and 20% independently by CH. The key inclusion criteria were that the study quantified distance or travel time to healthcare and identified whether there was an impact from this on health outcomes and the assessment of travel time/distance on the health outcome was the primary objective of the study.

10.1136/bmjopen-2016-013059.supp1 Supplementary data

The study excluded papers:

- Including children (<18 years and maternity).
- Reporting only patient opinions and views.
- Reporting only one off emergency events or travel by different types of emergency vehicles, including myocardial infarction, and transfers between healthcare facilities.
- Reporting only countries classed as global south.

The full papers of studies that met the inclusion criteria were reviewed by CK and CH, and data extraction and quality assessment were completed. Reference lists of included papers were then reviewed to identify any additional studies. These were subjected to the same review process described above. The quality assessment of the studies was undertaken using a modified version of the cohort studies, Critical Appraisal Skills Programme (CASP) tool^13^ linked to the PICO terms. It included key components of the CASP tool; for example, did the study address a clearly defined question? Had a representative population been used? Was the exposure (distance or travel time) accurately measured to minimise bias? And the same for the health outcome, whether potentially confounding variables had been identified and included in the analysis. In addition, we included whether the funding source was external to the organisation and whether the study was peer reviewed. This was important as studies completed in-house may have an inherent tendency to be biased. The data were extracted and assessed for quality by CK, according to the study protocol, and 20% were independently extracted and assessed by CH. No studies were excluded on the basis of the quality assessment.

## Results

**Figure 1:**
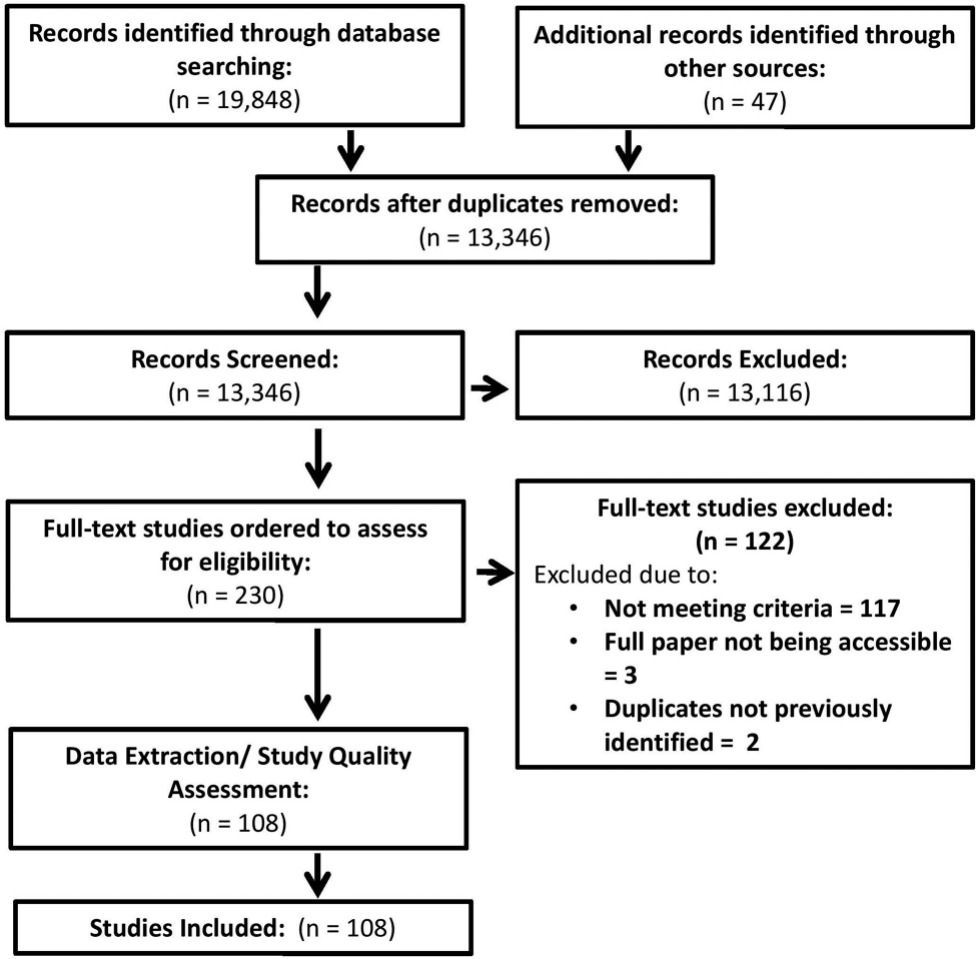
Flow diagram of papers.

One hundred and eight studies met the inclusion criteria and were included in the review. The study flow diagram is provided in figure 1, which shows that over 13 000 abstracts were initially reviewed. The studies covered a wide range of diseases, interventions and health outcomes. The results of the quality assessment are summarised in table 1. The main area of concern was the funding source of the study—37% of the studies were funded in-house or it was unclear how they were funded, which may lead to bias. However, no studies were excluded on the basis of this assessment.

**Table 1:** Quality assessment of studies n (%)

	Yes	No	Unclear/partial
Did the study address a clearly focused question?	108 (100%)	0	0
Was the study population recruited in an acceptable way?	105 (97.2%)	0	3 (2.8%)
Did it include all the population or describe the population not included?	97 (89.8%)	7 (6.5%)	4 (3.7%)
Was the method used to calculate the distance/travel time reported accurately?	85 (81.5%)	23 (18.5%)	0
Was the health outcome accurately measured to minimise bias?	108 (100%)	0	0
Have important confounding factors been taken account of in the design or analysis?	90 (83.3%)	17 (15.7%)	1 (1%)
Is the funding source external to the organisation?	68 (63.0%)	16 (14.8%)	24 (22.2%)
Was the research peer reviewed?	101 (93.5%)	0	7 (6.5%)

We have categorised the studies according to the following three groups:

1. *Distance decay association*—studies that showed evidence of an association between patients living closer to the healthcare facility and having better health outcomes/higher access rates to the healthcare services compared to those living further away (see online supplementary table S2).^7^ ^14–95^
2. *Distance bias association*—studies that showed evidence of an association between patients living further away from the healthcare facility and having better health outcomes/higher access rates to the healthcare services compared to those living closer to the healthcare facilities (see online supplementary table S3).^8^ ^96–100^
3. *No association*—those studies that found no evidence of an association between distance from the health facility and health outcome (see online supplementary table S4).^101–119^

10.1136/bmjopen-2016-013059.supp2 Supplementary tables

Seventy-seven per cent of the included studies identified a distance decay association; 6 studies reported a distance bias association and 19 identified no relationship.

The studies were diverse in nature; however, five of the distance bias studies (see online supplementary table S3) reported a positive relationship between increasing travel distance and better survival rates for patients with cancer.^96^ ^97^ ^98^ Lipe *et al*^99^ concluded that survival rates were higher for those travelling further to the transplant centre potentially due to referral bias, but also for patients living further away being healthier and more motivated. Other effects identified by the review include the study by Kim *et al*^42^ who highlighted a U-shaped all-cause mortality relationship. When the data were split into three categories of distance travelled, those in the middle (20–30 km) category had lower all-cause mortality than those living in the closer or further away categories. This indicated that there was something different about this geographical area and the people living in it. This effect was evidence in other papers, but not at statistically significant levels.

Over 50% of the studies reported on cancer (55% in online supplementary table S2, 83% in online supplementary table S3 and 53% in online supplementary table S4), with the majority being breast or colorectal studies. Other diseases and outcomes are summarised in online supplementary tables S2–4. The studies covered a wide range of contexts and travel requirements for patients. Studies that identified a distance decay association ranged from a very localised cohort of patients—average distances to the healthcare facility of 21.4 km for the treatment for diabetes,^71^ to >6 hours travel in Canada for breast and colorectal cancer survival,^25^ to >300 km for remote kidney dialysis^67^ and an intercountry study with a range of 1–870 km for treatment for malignant brain tumour.^41^ These differences reflect the geographical sizes of the countries in question and the need to travel for specialist treatment. There was no obvious difference in the distances and travel times between the three groups (distance decay, distance bias and no association).

A wide variety of methods and data (eg, registry data, patient surveys and hospital data) were used to explore the relationship. There were differences in the patient origins and healthcare destinations used to determine the patient journeys. The majority used the patients’ address (full address/postcode/zip code) as the origin for the journey, but others used the centroids of larger geographical areas^19^ ^34^ ^62^ ^90^ or the referring hospital^72^ or the city of residence.^116^ It was recognised that for the longitudinal studies, there was a potential for patients to move addresses, but no studies used differing residential locations where people moved house to calculate the distances and travel times. For example, Dejardin *et al*^27^ applied the residential location at the time of diagnosis and assumed this remained constant during treatment. Forty-eight per cent of the studies had access to data on the nearest healthcare facility to the patient, with the remainder using the actual healthcare facility attended. Bristow *et al*^20^ and Henry *et al*^107^ calculated the nearest and actual facility attended. All studies who found a distance bias association used the actual healthcare facility attended by the patients in their study.

The methods used for calculating travel distance/travel time in the studies ranged from straight-line distance (Euclidean distance), travel distance using a road network (either shortest distance or shortest travel time), travel speed using the shortest distance by road network (with and without adjusted road network speeds) or patients' self-reported travel times. As provided in table 1, 19% of the studies did not clearly state how they had calculated this variable. One hundred per cent of the studies in the distance bias association group calculated travel distance, 77% in the distance decay association group and 63% in the group that identified no association.

## Discussion

The results were mixed. Eighty-three studies identified evidence of distance decay association, 19 no evidence and 6 studies evidence of distance bias association. Thus, the majority of studies reported a negative correlation between distance/travel time to healthcare facilities and health outcomes. This was true across a multitude of disease groups, geographical distances and boundaries. The wide range of methods, sources of data, disease areas and outcome measures and ranges of distances travelled add to the complexity of the comparisons. The focus of this discussion is on the key differences in the way that the distances and travel times were calculated and analysed and what observations from the studies have heightened potential reasons to suggest an association between distance/travel time and health outcomes.

### Travelling to healthcare

The critical elements of calculating an accurate representation of the distances and travel times that the patients have endured require a starting location for the journey (eg, patients' home address),^i^ end point (healthcare facility) and method for accounting for the estimated route taken between these two points. The included studies differed on all three of these inputs. Where the patient's address was unavailable, less specific geographical identifiers were used by the studies, ranging from patients postcode,^91^ zip code centroid,^29^ centroid of a census district^62^ referral hospital,^72^ to the centroid of town of residence^116^ to a mixture of the above methods where data were missing at the less aggregated geographical levels.^101^ Using an origin point that is less accurate than the patient's home address has the potential to reduce the accuracy of the results, as it may influence the route taken affecting the distances and travel times.

The geographical data available for the healthcare facilities attended also differed across studies. Fifty-two per cent of the studies had the address of the healthcare facility attended by the patient. The remainder used the address of the nearest facility to the patient, as a proxy. Knowing how realistic the proxy measure is would be a benefit, as it may dramatically change the distances/travel times calculated. For example, Tracey *et al*^57^ identified in their study that only 37% of the patients attended the nearest facility, so using this as the proxy would underestimate the distances travelled by patients.

Another issue identified by the studies was that where patients were followed up over time—patients had the potential to move home address.^27^ ^59^ It was argued by some studies that grouping distances into large categorical bands allowed patients to move residence, but not actually move categories during the study (eg, Thompson *et al*,^65^ whereby 27% of the study's population changed their residence during the 5-year follow-up, but 91% of the patients had remained in the original distance category).

The majority of studies focused on one destination (eg, hospital attended), for one type of treatment (eg, an operation). This has the potential to underestimate the impact of distance/travel times on health outcomes—where patients are potentially making multiple trips to a range of hospitals over the course of the year for a range of health issues. In an attempt to be more representative of the travel burden, Brewer *et al*^19^ used the follow-up radiation centre address as the destination for patients rather than the place they had the surgery, as they argued patients would have to make this journey more frequently. Studies such as Jones *et al*^38^ considered the impact of a range of potential healthcare settings (eg, distance to the nearest cancer centre, general practitioner (GP) and hospital of first referral). They found a significant association between distance and survival for the GP, but not the other healthcare settings studied. Similarly, Wang *et al*^58^ found that as travel times to the nearest GP increased, patients were more likely to have a later stage breast cancer diagnosis, which was not evident when focusing on the distance to the nearest mammography service. These examples imply that focusing on a single site healthcare location (eg, hospital where the surgery took place) could be missing the location that most influenced the patient health outcomes.

### Measuring distance and travel time

Straight-line distance was used to calculate the distance for >25% of the studies. It is unlikely that any healthcare trip can be made in a straight line, but it was argued by some studies that grouping distances into categories that covered large geographical areas reduced the effects of differences between using road distance and straight-line distance. The remainder of the studies calculated travel time or road network-based distance (either shortest route or quickest route). This was calculated in a variety of ways, including making use of specific GIS software (eg, ESRI ArcGIS, MAPINFO and ARCinfo), but more recent papers had used online routing websites such as Google Maps, http://www.Mellisa.com or http://www.Mapquest.com. Online resources are straightforward to use and highly accessible to calculate distances and travel times, but there is a question as to whether patient data (eg, patients' home addresses and the hospital attended) should be uploaded to such websites and how secure this is, especially in the case of rarer diseases. A number of studies did take account of the time of year to control for potential differences in the weather and the impact this might have,^101^ but none included traffic congestion to calculate the travel times, which could significantly have increased the travel times included.

Distances and travel times were included in the statistical models as continuous or categorical variables or both separately. Studies identified that distances/travel times tended to be positively skewed towards more patients living closer to the healthcare facilities that they were attending. To better represent this phenomenon, Haynes *et al*^34^ split the travel times into categories according to the lowest quartile, medium (quartiles 2 and 3), high (75–95th centile) and highest (95–100th centile) categories. Other studies linearised distance/travel time from the natural scale to the log scale, but the majority did not. For studies that included distance/travel times as a categorical variable, there was no consensus on what categories should be used. Study examples include Sauerzapf *et al*^111^ who split the travel distances into <30, 30–60 and >60 miles, Panagopoulou *et al*^49^ used dichotomous categories < 300 and > 300 km, Littenberg *et al*^69^ split data into < 10 and ≥ 10 km, and Allen *et al*^82^ calculated the mean distance and used this to split the data into two groups. Other studies used quartiles or quintiles. In many cases, no justification was given for how the categories were determined, which has the potential to hide effects, where critical thresholds are missed. What the studies did identify was that the results were sensitive to the cut-offs used in the model. Athas *et al*^17^ found that after adjusting for age, the likelihood of receiving radiotherapy following breast-conserving surgery decreased significantly with increasing travel distance to the nearest facility for distances >74 compared to <10 miles, but not for categories in-between. In this case, a dichotomous threshold that compared <30 and ≥30 might not have picked up this effect. Studies may be advised to undertake sensitivity analysis around the reference distance groups and categories used in their models—as this may greatly influence the results. Abou-Nassar *et al*^14^ and Maheswaran *et al*^45^ presented results that were only significant in the model that treated distance as a continuous variable; again, the categories might not have been sensitive enough to pick up any effect.

### Mode of transport

It was assumed in the majority of studies that patients would travel by car although there were exceptions.^81^ ^83^ ^64^ For some patients (potentially in the most deprived groups), it will not be possible to access healthcare by car. Moist *et al*^64^ reported that increased public transport travel time for patients contributed to missing kidney dialysis sessions. Jennings *et al*^76^ reported that public transport travel times were longer for patients who did not attend follow-up appointments compared to those who did. Other studies included public transport access through proxy measures (eg, whether patients were within 800 m walking distance of an hourly bus service). Issues with this include that it does not account for whether the bus service identified goes to the hospital, the travel time once on the bus or the likelihood of the patient being able to walk 800 m. In one study, a travel survey of patients' trips to the hospital found that 87% were made by car.^103^ To ensure representative travel times/distance, it is critical to understand the patient population (in this case how they are travelling).

### Key relationships

The studies in the review highlight some of the key factors that were found to be more sensitive to the distance decay effect. For example, Joseph and Boeckh^80^ identified that the distance decay effect was more pronounced for less serious illnesses and Arcury *et al*^83^ identified that patients attended significantly more regular check-up care visits the shorter the distance to the facility. While, for Lara *et al*,^77^ distance was a predictive factor for not attending *in-between* follow-up appointments (6 and 9 months), whereas it was not predictive for the 12-month or 3-month follow-up appointments following a gastric band being fitted. These studies all suggest that when patients feel the health situation is more serious or they live closer they are more likely to attend. In their study, Abou-Nassar *et al*^14^ found that the impact of distance on health outcomes was only significant 1 year after a transplant, suggesting that the point at which the health outcome and distance is measured could be critical to the results. Lake *et al*^91^ identified that while there was an effect of distance on patients attending treatment for tuberculosis (TB), when doing subgroup analysis this was only significant for those patients not native to the country, so potentially identifying an impact of reduced ability to travel for patients who are less familiar with the healthcare system and transport network. All of which could be considered when tailoring healthcare provision and require further research.

One of the key influencing variables identified by the studies was deprivation. Dejardin *et al*^27^ found that when controlling for deprivation that the effect of distance on health outcomes was removed, whereas Crawford *et al*^26^ observed that distance amplified the effect from deprivation. From one side, it might be argued that by controlling for deprivation, this is also removing some of the impact of distance/time that is experienced by those who do not have access to a car and would have to travel by other means. For those studies in the review, not controlling for deprivation may be overestimating the true impact of distance travelled/travel time on patient's health.

Studies such as those in online supplementary table S3 (distance bias association) show that, in some cases, patients are capable of travelling longer distances and have better health outcomes than those living closer. This indicates that there are factors other than distance (such as deprivation) that are contributing to how easily patients can travel to access the healthcare facilities. Differences in distances that patients would be willing to travel (travel thresholds) to the primary care practice have been explored in studies such as McGrail *et al*^120^ who asked patients “what would be the maximum distance they would be willing to travel to access their GP?” (for a non-emergency). Communities where the population was sparsely located were found to be willing to travel a maximum of 22.2 minutes more to visit the primary care practice than those in closely settled communities. Buzza *et al* found that distance was the most important barrier to accessing healthcare in their study, but also identified “health status, functional impairment, travel costs and work or family obligation” as key barriers (ref. 121, p. 648). Similarly, the Social Exclusion Unit (SEU) in the UK proposed that a person's ability to travel was influenced by key factors, including their *travel horizons* (where are they willing to travel to?, what maximum distance? and do they have full awareness of available transport options for the journey), *Cost* (can they afford to travel to the healthcare facility?), *Physical Access* (their health state may make accessing transport physically difficult or if accessing public transport, there may not be an appropriate route) and *Crime* (they may not want to travel unless they felt safe making the journey) SEU.^122^ All these factors need to be considered when focusing on where to locate a healthcare facility/improve access for patients to an existing facility and ultimately improve health outcomes. For studies such as Bristow *et al*,^96^ closer investigation of those patients living, <5 km from the hospital whose health outcomes were worse than those living further away, or in the case of Kim *et al*^42^ what makes those patients living 20–30 km away have better health outcomes—what makes them different? And how can these other groups be better supported to access healthcare services? Using the types of studies brought together in this review allows some of these questions to be explored and inform debate over potential solutions.

The reason for undertaking this review was to collate and review evidence on the potential impact of distance and travel time to healthcare on patients' health outcomes. This is particularly pertinent given the move to centralised specialist services, which typically mean increased travel distance to access those healthcare facilities. Studies such as Kerschbaumer *et al*^41^ have shown that if follow-up can be completed successfully at a local level (even if the surgery is centralised), this can improve health outcomes and reduced travel burden. The review has shown that by making use of ex-post healthcare data, providers can identify spatially pockets of patients who would be disadvantaged through having to travel further to access healthcare facilities and could use this to examine how these patients match with existing support and transport networks. It has also shown that it is not just about identifying patients who have to travel the furthest with evidence of patients living in close proximity to the healthcare facilities often fairing the worst. More research is needed to pick up on these factors and to explore in more detail the impact that the methods and data sources have on the results.

### Strengths and limitations

This systematic review has, for the first time, synthesised available evidence on the association between differences in travel time/distance to healthcare services and patient's health outcomes. It has identified a wealth of studies and generated evidence for wide range of disease groups and health outcomes, across multiple countries. There was great variation in study design, distances and travel times to the healthcare setting, and the range of health outcomes; this precluded pooling of data for meta-analysis. The study followed a search strategy to maximise the identification of relevant studies; of which, 19 did not find an association between distance/travel time and health outcomes; this is likely to be an underrepresentation if authors have a tendency to not publish results that showed no effect. While the review findings are of undoubted value in broadening our understanding of the wider societal factors that influence health outcomes, their applicability may be limited to countries with similar healthcare systems.

## Conclusions

In the debate between local versus centralised healthcare provision, 77% of the included studies showed evidence of an association between worse health outcomes the further a patient lived from the healthcare facilities they needed to attend. This was evident at all levels of geography—local level, interurban and intercountry level. A distance decay effect cannot be ruled out, and distance/travel time should be a consideration when configuring the locations of healthcare facilities and treatment options for patients.

## Footnotes

i It is noted that not all patient journeys start from the patient's home address. This is, therefore, a proxy measure.
