# Supplementary material for: Are differences in travel time or distance to healthcare for adults in global north countries associated with an impact on health outcomes? A systematic review
Source: BMJ Open. 2016 Nov 24;6(11):e013059. doi: 10.1136/bmjopen-2016-013059 (PMC5178808; doi:10.1136/bmjopen-2016-013059)
Supplement: Supplementary data [file bmjopen-2016-013059supp.pdf]

Supplementary file 1: Search Terms for MEDLINE

**Intervention/ Comparator terms**	**Population accessing Healthcare**	**Health Outcomes**
Proximity adj3 health*.ti,ab	health*adj3 access*.ti,ab	Health status.ab,ti
Proximity adj3 hospital*.ti,ab	health* adj3 care.ti,ab	Health inequal*.ab,ti
Travel*.ab,ti	health* adj3 facilit*.ti,ab	"health related quality of life".ab,ti
Distance*.ab,ti	hospital*.ti,ab	Hrqol.ab,ti
Patient adj3 transport.ti,ab	inpatient*.ab,ti	Mortality.ab,ti
Journey*adj5 (car or bus or transit or transport* or public transport or train).ti,ab	outpatient*.ti.ab	Delay* adj3 diagnosis.ab,ti
Time to hospital*.ab,ti	health* adj3 appoint*.ab,ti	Late* adj3 diagnosis.ab,ti
Transportation of patients/	rural adj3 health*.ab.ti	Miss*adj3 appoint*.ab,ti
Travel/	urban adj3 health*.ab,ti	Health adj3 outcome.ab,ti
	communit* adj3 health*.ti,ab	Quality of life.ab,ti
	primary health*.ab,ti	Self reported health.ab,ti
	family practice.ab,ti	Prognosis.ab,ti
	gen* pract*.ab,ti	Complete adj3 treatment.ab,ti
	health* adj3 screen*.ti,ab	Did not attend.ab,ti
	clinic.ab,ti or clinics.ab,ti	Health status/ or health status disparities/
	GP.ab,ti	*"Quality of life"/ or patient compliance/ or patient refusal/ or diagnosis/ or delayed diagnosis/
	"accident and emergency".ab,ti	Mortality/
	health services accessibility/	Prognosis/

	hospitals/ or hospitals, community/ or hospitals, general/ or hospitals, group practice/ or hospitals, high-volume/ or hospitals, low-volume/ or hospitals, private/ or hospitals, public/ or hospitals, rural/ or hospitals, satellite/ or hospitals, special/ or hospitals, teaching/ or hospitals, urban/ or mobile health units/ or secondary care centers/ or tertiary care centers/Appointments and schedules/	Treatment adj3 retention.ab,ti
	Mass screening/	Treatment adj3 follow adj3 up.ab,ti
	Urban health/	Patient complian*.ab,ti
	Rural health/	
	Health services/ or primary healthcare/ or general practice/ or tertiary healthcare/	
	Emergency service, hospital/	

Restrictions	NOT exercise test/ or exercise test.ab,ti
	English Language
