# Supplementary material for: Are differences in travel time or distance to healthcare for adults in global north countries associated with an impact on health outcomes? A systematic review
Source: BMJ Open. 2016 Nov 24;6(11):e013059. doi: 10.1136/bmjopen-2016-013059 (PMC5178808; doi:10.1136/bmjopen-2016-013059)
Supplement: Supplementary tables [file bmjopen-2016-013059supp_tables.pdf]

## Supplement table 2: Included studies that identified evidence of a distance bias association

| Author<br><br>Country<br><br>Date | Disease / Procedure | Source, Years & Sample size | Health Outcome | Distance/ travel time measurement | Origin and Destination | Summary of key results |
|---|---|---|---|---|---|---|
| **Cancer Studies** | | | | | | |
| Abou-Nassar, et al. [14]<br><br>USA<br><br>2012 | Allogeneic Hematopoietic Stem Cell Transplantation | Clinical Operations and Research Information Systems database at DF/BWCC.<br><br>1996 - 2009.<br><br>Sample = 1,912 (meeting the criteria of living < 6 hours to the treatment centre). | Overall Survival | Travel Time.<br><br>Calculated using driving distance and average driving speeds along the road network<br><br>Travel time was treated as a categorical variable using 3 groups: ≤40, 41 - 159, ≥160 mins and also a continuous variable. The range of distances was 2 - 358 mins. | Patients' Residence<br><br>TO<br><br>The transplant Centre | The study found that longer drive times to the transplant centres was associated with worse overall survival in patients alive and disease free after 1 year - This was only true using travel time as a continuous variable. They suggest this may be in part related to the lower number of visits in patients living further away after receiving the transplant. |
| Albornoz et al. [15]<br><br>USA<br><br>2015 | Breast Reconstruction | National Cancer Database<br><br>Included Patients who had a unilateral or bilateral mastectomy with or without reconstruction 1998 – 2011<br><br>1,031,343 | The rate and method of breast reconstruction services | Straight-line Distance<br><br>Straight-line distance. Using the "Great Circle Distance" in the database.<br><br>Treated as a continuous variable.<br><br>For 2011 the average distance travelled for mastectomy without reconstruction – 27.1 miles and 34 miles with reconstruction. | Patients' Residence (zip code or city if zip code was unavailable)<br><br>TO<br><br>Hospital that reported the case. | The study found that patents had travelled further for breast reconstruction services than for mastectomy without reconstruction. Indicating a distance bias. Patients were more likely to have immediate breast reconstruction the further they had travelled (0-20 miles 13.9% reconstruction 101-201 24.9%). |
| Anderson, et al. [16]<br><br>USA<br><br>2013 | Colorectal Cancer | A set of cross sectional telephone survey of the population > 18 years in the USA. Taken from the Utah Behaviour Risk Factor Surveillance System.<br><br>2010 | Adherence to risk appropriate screening guidelines | Travel Time<br><br>The study calculated 1 mile grid cells for the state of Utah and for each grid cell populated with individuals aged 50 or older they calculated the actual travel time to the nearest colonoscopy provider. This was then used to | Patients' Residence (determined using a 1 mile grid reference for the addresses)<br><br>TO<br><br>The nearest colonoscopy provider. | The study found that residents living > 20 mins from the nearest colonoscopy provider were significantly less likely to be up-to-date with risk appropriate screening than those living < 10 mins from the nearest provider. |

| | | | | | | |
|---|---|---|---|---|---|---|
| | | Sample = 2,844 | | calculate a population weighted median travel time by zip code.<br><br>Travel times was treated as a categorical variable and grouped into 3 categories: <10 minutes, 10 - 20 minutes & >20 minutes. | | |
| Athas, et al. [17]<br><br>USA<br><br>1999 | Breast Cancer | New Mexico Tumour Registry & The National Cancer Institute's surveillance Epidemiology and End Results.<br><br>Patient Diagnosed 1994 – 1995<br><br>Sample = 1,122 | Receipt of radiotherapy following breast conserving surgery | Straight-Line Distance.<br><br>Distance was treated as a categorical variable and split into the following categories: <10 miles, 10.0-24.9, 25.0-49.9, 50.0-74.9, 75.0-99.9, ≥100 miles. | Patients' Residence (street address (70% of cases) and centroid of residential zip codes (30%)).<br><br>TO<br><br>The nearest radiation treatment facility. | The study found that by controlling for age the likelihood of receiving radiotherapy following breast conserving surgery decreased significantly with increasing travel distance to the nearest facility. This was significant for distances >74.9miles compared to a base of <10miles. |
| Baade, et al. [18]<br><br>AUSTRALIA<br><br>2011 | Rectal Cancer | Queensland Cancer Registry (QCR)<br><br>1996 - 2007<br><br>Sample = 6,848 | Cause specific survival | Road Distance and Travel Times.<br><br>The distances were treated as a categorical variable using the following groups: < 50km, 50 - 99km, 100 - 199, 200 – 399 and ≥400km.<br>The travel times were treated as a categorical variable using the categories of 0 -1hours, 2-4, 4-6, ≥ 6 hours | Patients' Residence<br><br>TO<br><br>The nearest radiotherapy facility | The study found that after adjusting for age, sex and stage at diagnosis, patients who lived 100 - 199km, 200-399km and 400km or more from a radiotherapy facility were 16%, 30% and 25% respectively more likely to die from cancer than patients living within 50km of such a facility. For every 100km increase in distance there was on average a 6% increase in risk of mortality. Similar results were found when travel time was used in the calculations, where patients living greater than 6 hours away were 22% more likely to die from cancer than those living 0- 1 hours away. |
| Brewer, et al. [19]<br><br>NEW ZEALAND<br><br>2012 | Cervical Cancer | New Zealand Cancer Registry.<br><br>1994 - 2005<br><br>Sample = 1,383 | Cancer screening, stage at diagnosis and mortality | Travel Time and Road distance.<br><br>The distances and travel times were treated as categorical variables using the following method of grouping - low - the lowest quartile, Medium - quartiles 2 and 3, High - records between the 75th and 95th percentiles and Highest - the highest 5% of records. | The 2001 census area unit for the patient (population weighted centroid)<br><br>TO<br><br>The nearest GP and nearest Cancer Centre | The study found that increased travel time/ distance was weakly associated with cervical cancer screening, stage at diagnosis and mortality. |

| | | | | | | |
|---|---|---|---|---|---|---|
| Bristow, et al. [20]<br><br>USA<br><br>2014 | Ovarian Cancer | Californian Cancer Registry<br><br>1996 - 2006<br><br>Sample = 11,770 | Treatment Adherence | Distance.<br><br>(Does not say what method used) calculated using ESRI ArcMAP<br><br>Distance was treated as a categorical variable and split into quintiles from < 5km up to > 80km. | Patients' Residence<br><br>TO<br><br>The treating hospital and the closest high volume hospital. | The study found that living > 80km (compared to < 9km) from a high volume hospital was associated with an increased risk of non-adherence to care plans (OR = 1.88, Confidence interval, 1.61 - 2.10).<br><br>The study found that distance to a high volume hospital and distance to receive treatment could be used to predict whether patients would meet the guidelines for care for advanced stage ovarian cancer. |
| Burmeister, et al. [21]<br><br>AUSTRALIA<br><br>2010 | Lung Cancer | Queensland Cancer Registry.<br><br>2000 - 2004<br>Sample = 1,535 | Delay in receiving radiation therapy Survival | Road Distance.<br><br>(no info on GIS methods used) Distance was treated as a categorical variable using the groups of < 50km (where it was assumed that patients could travel on a daily basis from home) 50 - 200km (where it was assumed patients would go home for weekends only) and > 200km (where it was assumed that patients would need to spend the duration of their treatment at the hospital). | Patients' Residence (postcode)<br><br>TO<br><br>The nearest public radiation treatment facility. | The study found that waiting times for radiation therapy among lung cancer patients in Queensland was not associated with distance from home to the nearest public radiation treatment facility. But the study did find that those living > 200km away had slightly worse survival than those who lived < 50km. |
| Campbell, et al. [22]<br><br>UK<br><br>2001 | Colorectal and Lung Cancer | Scottish Cancer Registry<br><br>1995 - 1996<br><br>Sample = 1,323 | Presence of disseminated disease at diagnosis & emergency presentation or surgery. | Straight-line Distance.<br><br>Distance was treated as a categorical variable using the groups of 0 - 5km, 6 - 37km, 38 - 57km and ≥58km. These were pre-defined cut off points. | Patients' Residence - (Census output area centroids)<br><br>TO<br><br>The nearest cancer centre. | The study identified that increasing distance from the nearest cancer centre was associated with a higher chance of disseminated disease at diagnosis and therefore lower chances of survival. |
| Campbell, et al. [23]<br><br>UK<br><br>2000 | Cancer (Lung, Colorectal, Breast, Stomach, Prostate, Ovary) | Scottish Cancer Registry<br><br>1991 - 1995<br><br>Sample = 63,976 | One Year Survival | Straight-line Distance.<br><br>Distance was treated as a categorical variable using the groups ≤ 5km, 6 - 13km, 14 - 23km, 24-37km and ≥38km. | Patients' Residence (postcode)<br><br>TO<br><br>The nearest cancer centre | The study found that increasing distance from the nearest cancer centre was associated with a reduced chance of diagnosis before death for stomach, breast and colorectal cancer and poorer survival after diagnosis for prostate and lung cancer. |

| | | | | | | |
|---|---|---|---|---|---|---|
| Celaya, et al. [24]<br><br>USA<br><br>2006 | Breast Cancer | New Hampshire State Cancer Registry.<br><br>1998 - 2001.<br><br>Sample = 2,861 | Type of treatment received - either breast conserving surgery with radiography or Mastectomy | Straight-line Distance.<br><br>Distances were treated as categorical variable using the groups <20 miles, 20 to <40, 40 to < 60, ≥60 miles. The mean distance was 15.1 miles (range 0.1–89.9). | Patients' Residence (Residential Address geocoded (80%) or zip code centroid (20%))<br><br>TO<br><br>The nearest radiation treatment facility. | The study found that women were less likely to have breast conserving surgery with increasing distance from the nearest facility.<br><br>They were also less likely to have radiation therapy the further away they lived - if they had previously undergone breast conserving surgery. |
| Cramb, et al. [25]<br><br>AUSTRALIA<br><br>2012 | Breast Cancer and colorectal cancer | Queensland Cancer Registry.<br><br>1996 - 2007<br><br>Sample = 26,390 Males = 14,690 and Females = 11,700 | Survival and premature deaths | Travel Time.<br><br>Shortest travelling time by road. Travel time was grouped into 3 categories based on practical considerations. < 2hours, 2 - 6 hours and >6 hours | Centroid of the patients' statistical local area<br><br>TO<br><br>The closest radiation facility | The study concluded that the proportion of premature deaths was higher for those living >2 hours from a treatment facility for breast cancer. Colorectal patients living > 6 hours from a treatment facility had poorer outcomes than those in the 2- 6 hour category, but this was not statistically significant. |
| Crawford et al. [26]<br><br>UK<br><br>2009 | Lung Cancer | Northern and Yorkshire Cancer Registry and Information Service.<br><br>1994 - 2002<br><br>Sample = 34,923 | Diagnosis and form of treatment | Travel Time.<br><br>Calculated using ArcGIS 9.2 using average car speeds along the shortest route.<br><br>Travel time was treated as a categorical variable - dividing the patients into equal quartiles. Patients were then put into 1/ 16 groups that combined 4 quartiles of travel time and 4 quartiles of deprivation. | Patients' Residence<br><br>TO<br><br>The closest hospital providing diagnostic access. | The study found that patients living in the most deprived areas were least likely to receive histological diagnosis, active treatment and thoracic surgery. They found that travel time "amplified this effect "– patients in the most distant & most deprived group had the worst outcomes. |
| Dejardin, et al. [27]<br><br>FRANCE & ENGLAND<br><br>2014 | Colorectal cancer | 3 Cancer registries (Calvados, Cote d'Or and Saone et Loire) and 1 cancer registry in England (Northern and Yorkshire).<br><br>1997 - 2004<br><br>Sample = 40,613 | Survival | Travel Time.<br><br>Using ArcGIS in England and Mapinfo in France. The study used road map databases using legal speed limits by road class.<br><br>Travel time was treated as a categorical variable using the 5 groups of 0 - 5 mins, 6 - 20 mins, 21 - 40mins , 41 - 90 mins and ≥ 91mins for travel times to the | Patients' Residence (at the time of diagnosis)<br><br>TO<br><br>The nearest cancer centre, radiotherapy centre and hospital. | The study identified (unadjusted analysis) that travel times were significantly associated with survival, as patients living further from healthcare resources had a better chance of survival than those living closer. When including material deprivation in the model this effect was removed. |

| | | | | | | |
|---|---|---|---|---|---|---|
| | | | | nearest cancer centre & nearest radiotherapy unit and 0 - 5, 6 - 10, 11-15, 16 - 40 and ≥41mins for travel to the nearest hospital. | | |
| Dupont-Lucas, et al. [28]<br><br>FRANCE<br><br>2011 | Colorectal Cancer | Clinical trials in Calvados Normandy and Cote-d'Or Burgundy - testing the diagnostic properties of two types of faecal occult blood test.<br><br>June 2004 - December 2006<br><br>Sample = 4,131 | Colonoscopy uptake | Road Distances.<br><br>Calculated using Mapinfo 9.1 combined with CHRONOMAP 2.1 based on the MultiNet Map database (Tele Atlas).<br><br>Distances were grouped into quartiles: 0 - 5.5km, 5.5 - 13.8, 13.8 - 22.1 & 22.1 - 52.3km. | Patients' Residence ( Home Address )<br><br>TO<br><br>The nearest gastroenterologist / or regional capital /or clinical trial centre | The study found that distance to the regional capital and distance to the clinical trial centre were independently associated with colonoscopy uptake. Distance to the nearest gastroenterologist was not found to be significant. |
| Engelman, et al. [29]<br><br>USA<br><br>2002 | Breast Cancer | The Health Care Financing Administration enrolment database to identify each fee for service Medicare eligible women in Kansas. - Medicare Claims data.<br><br>1997 - 1998<br><br>Sample = 117,901 | Mammogram attendance | Straight-line Distance.<br><br>Distance was treated as a continuous variable. | Patients' Residence (zip code)<br><br>TO<br><br>The nearest permanent & mobile mammography sites. | The study showed that increasing distance from a permanent mammogram facility was significantly associated with decreased mammogram rates. After controlling for age, race and education this relationship was still significant. OR = 0.97 for each 5 mile increment. |
| Fournel, et al. [30]<br>FRANCE<br><br>2010 | Colorectal Cancer | Burgundy Registry.<br><br>1990 - 1999.<br><br>Sample = 6220 colorectal adenoma patients and 2,387 colorectal cancer patients. | Colorectal adenoma detection | Distance.<br><br>(method not reported)<br>Distance were included as a categorical variable using groupings of <5km, 5 - 15km and >15km. | Patients' Residence<br><br>TO<br><br>The GP, hepatogastroenterologist (HGE), and physician (not clear whether these were the nearest) | The study found that incidences of colorectal cancer were not significantly associated with distance to the GP, HGE, or the physician. The study did find a significant interaction between place of residence and the distance to the GP and place of residence and the HGE. The impact of the distance to the physicians was significant for patients living in rural areas. |
| Giuliani et al. [31]<br><br><br>Italy | Breast Cancer | Romagna Cancer Registry<br><br>Patients were included | Compliance with yearly mammography and /or Clinical | Travel Times.<br><br>Calculated using Google Maps.<br><br>Travel time was split into | Patients' Residence (assumed not stated)<br><br>TO | The study found that patients were less likely to have a yearly check-up (over the 10 years) if they had to travel >30 mins compared to ≤15 mins. |

| | | | | | | |
|---|---|---|---|---|---|---|
| 2016 | | if they had a diagnosis of in situ and invasive cancer between<br>1990 – 2000<br>735 | breast examination over 10 year follow up period. | categories ≤15 mins, 16 – 30 and >30.<br>The study also considered the altitude of the patient's residence. | The centre for cancer prevention | |
| Goyal et al. [32]<br>USA<br>2015 | Breast Cancer | Breast Cancer Disparity Cohort Study (New Jersey)<br>African American and white patients diagnosed with early stage breast cancer.<br>2005 - 2011<br>623 | Mastectomy OR Breast conserving surgery followed by adjuvant radiation therapy | Travel Distance and Travel Time<br>Shortest travel time/ distance was calculated using Google Maps.<br>Distance and travel times were treated as categorical variables and split up into quartiles.<br>Travel distance <3.2miles, 3.2-5.6, 5.7-9.2 and >9.2miles.<br>Travel times <9 mins, 9-13 mins, 14-19 mins and >19 mins. | Patients' Residence<br>TO<br>The radiation facility where patients received Radiation Therapy (where unavailable-surgeons were contacted by phone and the referral obtained) | The study found that patients living further away from the radiation therapy centre in the categories of 5.7-9.2miles and >9.2miles compared to < 3.2 miles (REF) were significantly more likely to have a mastectomy than breast conserving surgery followed by RT.<br>Patients living > 19mins compared to <9 mins were also more likely to receive a mastectomy rather than breast conserving surgery. |
| Haddad et al. [33]<br>USA<br>2015 | Bladder Cancer | Urban tertiary cancer centre (single site)<br>2007 – 2013<br>406 | Short and long term survival after radical cystectomy | Shortest Driving Distance<br>Calculated using Google Maps<br>Distance was treated as a categorical variable. Using the categories of < 50 miles, 50 – 100, 100.1 – 150 and >150 miles.<br>Median distance 37.3miles | Patients' Residence<br>TO<br>The Treatment Facility (Single Site) | The study found that increasing distance to the facility was a significant predictor of 90 day mortality (univariate model) and was still significant after controlling for nodal status. For long term survival distance was significant for those travelling >150miles versus <50miles for the univariate model. |

| | | | | | | |
|---|---|---|---|---|---|---|
| Haynes, et al. [34]<br><br>New Zealand<br><br>2008 | Cancer (prostate, colorectal, breast, lung, melanoma) | New Zealand Ministry of Health<br><br>1994 - 2006<br><br>Sample = 1,383 | Late diagnosis and likelihood of death | Travel Time.<br><br>Travel time was treated as a categorical variable and split into 4 categories (Low, medium, High, Highest) low - lowest quartile, medium (quartile 2 and 3) high records between 75% and 95 percentiles and highest - highest 5% of records. This grouping was used to account for the skewed travel times. | Population weighted centroid of the 2001 census area units (CAU represent approx. 2300 people)<br><br>TO<br><br>The nearest cancer centre and nearest GP | The study had mixed results. After controlling for the extent of the disease, poor survival was associated with longer travel times to the GP for prostate cancer and longer travel times to the nearest cancer centre for colorectal, breast and prostate cancers, but not lung cancer or melanoma The study found that the disease tended to be less advanced in patients who lived further from the cancer centres and living further from a GP practice was not associated with a later stage diagnosis. |
| Holmes, et al. [35]<br><br>USA<br><br>2012 | Prostate Cancer | Physician workforce study in North Carolina & North Carolina Central Cancer Registry on patients diagnosed with incident cancer linked to Medicare claims.<br><br>2004 - 2005<br><br>Sample = 2,251 | Delayed Diagnosis | Straight-line Distance.<br><br>Distance was treated as a categorical variable and used 3 groups of: 0 - 10 miles, 11 - 20miles and > 20 miles. | Patients' Residence ( zip code centroid of patient residence)<br><br>TO<br><br>The nearest urologist | The study found that increasing distance to an urologist was significantly associated with higher risk of prostate cancer at diagnosis, which was higher for black patients. |
| Huang, et al. [36]<br><br>USA<br><br>2009 | Breast Cancer | Kentucky Cancer Registry.<br><br>1999 - 2003<br><br>Sample = 12,322 | Diagnosis Stage | Road Distance.<br><br>Distance was treated as a categorical variable using the groups - <5 miles, 5 - 9, 10 - 14 and ≥15 miles | Patients' Residence ( 78% were geocoded based on street address. 15% using the centroid of the 5 digit zip code and 7% using the 5 digit zip code + 2 or + 4 digits)<br><br>TO<br><br>The nearest mammogram centre | The study found that patients diagnosed with advanced stage diagnosis had longer average travel distances than early stage diagnosis. After controlling for age, race, insurance and education the odds of advanced diagnosis were significantly greater for women living ≥15 miles compared to those living <5 miles. |

| | | | | | | |
|---|---|---|---|---|---|---|
| Jethwa, et al. [37]<br><br>USA<br><br>2013 | Breast Cancer | Hospital Records.<br><br>2007<br><br>Sample = 260 (women were excluded if they were non-white or had a previous cancer diagnosis) | Stage of breast cancer at diagnosis, survival | Distance.<br><br>(Unknown calculation). Distance was treated as a categorical variable using the following groups: < 15 miles, 15 - 44 miles, 45 - 59 miles, and ≥60 miles. | Patients' Residence<br><br>TO<br><br>The treating hospital | The study found that the further the distance the more likely women were to be diagnosed at a later stage and the more likely women were to have a mastectomy.<br><br>The study found no association between travel distance, age at diagnosis, receipt of radiotherapy, or 5-year survival. |
| Jones, et al. [38]<br>UK<br><br>2008 | Breast colorectal, lung, ovarian and prostate cancer | Northern and Yorkshire Cancer Registry and Information Service (NYCRIS)<br><br>1994 - 2002<br><br>Sample = 117,097 | Survival (whether patients were alive or dead on 31st March 2005) and late stage diagnosis | Travel Times.<br><br>Calculated using average car travel speeds by road class on the road network. Travel time was treated as a continuous variable.<br><br>The study also determined: - whether patients were within 800m of an hourly bus service for rural patients. Straight-line distance to the nearest cancer centre, car journey to the closest railway station, travel time to the GP and first referral hospital. | Patients' Residence<br><br>TO<br><br>The GP, Hospital of first referral and closest cancer centre | The study found that late stage diagnosis was associated with increasing travel time to the GP for breast and colorectal cancer and risk of death was associated with increased travel time to the GP for prostate cancer.<br><br>The study identified residential deprivation was significantly related to survival. |
| Jones, et al. [39]<br><br>UK<br><br>2010 | Cancer (Colorectal, ovary, breast, prostate) | Northern and Yorkshire Cancer Registry Information Service.<br><br>1994 - 2002.<br><br>Sample = 3,536 | Whether or not the diagnosis was made at death. (Diagnosis date = death date) | Road Distance and Travel time<br><br>Estimated using average travel speeds over the road network. The study also calculated straight-line distance and assessed whether patients lived within 800m walking distance of an hourly weekday bus service & whether there was a local community transport scheme. Travel time to hospital was modelled as a categorical variable using quartiles. | Patients' Residence (postcode)<br><br>TO<br><br>The nearest healthcare provider postcode/ Nearest GP | The study found that the highest odds of being diagnosed at death were for those living in the least accessible quartile of travel time for the hospital, but this association was only statistically significant for colorectal and ovary cancer.<br><br>The study found that living in the least accessible travel time quartile to the GP had the highest odds of being diagnosed at death, but was not statistically significance.<br><br>Breast and prostate cancer patients living closer to a frequent bus service were significantly less likely to be diagnosed at death. |

| | | | | | | |
|---|---|---|---|---|---|---|
| Jones, et al. [40]<br><br>UK<br><br>2008 | Breast, Colon, Rectum, Lung, Ovary and Prostate Cancer | Northern and Yorkshire Cancer Registry (NYCRIS)<br><br>1994 - 2002<br><br>Sample = 117,097 | Patients receiving surgery, chemotherapy or radiotherapy | Travel Time.<br><br>Travel time was modelled as a categorical variable and divided into quartiles. | Patients' Residence (home postcode)<br><br>TO<br><br>The nearest hospitals providing treatment. | The study identified an inverse relationship between travel time and treatment take up. Patients were less likely to receive radiotherapy the further they lived from the hospital.<br>Lung cancer patients were less likely to receive surgery & Lung and rectal patients were less likely to receive chemotherapy. |
| Kerschbaumer, et al. [41]<br><br>AUSTRIA<br><br>2012 | Glioblastoma Multiforme (GBM) - malignant brain tumor | Medical Records<br><br>1990 - 2009<br><br>Sample = 208 | Survival (Months) | Shortest Road Distance.<br><br>Distance was treated as a continuous variable. Average distance was 75km (range 1 - 870km) | Patients' Residence (home address)<br><br>TO<br><br>The neuro oncological centre | The study found that distance to the neuro oncological centre had a significant effect on overall survival. Patients were less likely to be treated with chemotherapy following surgery the further they lived away from the centre. The study found that when a new treatment was introduced that could be administered locally this removed this effect. |
| Kim, et al. [42]<br><br>UK<br><br>2000 | Colorectal cancer | South and West Cancer Intelligence unit.<br><br>1991 - 1995<br><br>Sample = 4,962 | Survival | Straight-line Distance.<br><br>Distance was treated as a categorical variable using the following groups - ≤10 km, > 10 to ≤ 20 km, > 20 to ≤ 30 km and > 30km. | Patients' Residence(postcode)<br><br>TO<br><br>The treating hospital | The study found that those travelling ≥ 30km from the treating hospital had significantly poorer survival, but that those living 20 - 30 km away appeared to be least at risk. Implying a U shape in terms of risk. |
| Lavergne, et al. [43]<br><br>CANADA<br><br>2011 | Palliative Radiotherapy (PRT)- Cancer | Oncology Patient Information System (Nova Scotia)<br><br>2000-2005<br><br>Sample = 13,494 | PRT Treatment & Consultation | Travel Time.<br><br>Calculated using "GIS" and average vehicle speeds by road type.<br><br>Travel time was treated as a categorical variable using 4 categories: 0 - <30 mins, 30 - < 60 mins, 60 - < 120 mins and 120 - 214mins. | Patients' Residence (postcode at death)<br><br>TO<br><br>The nearest treatment centre | The study found that Palliative radiotherapy use declined with increasing travel time and community deprivation. |
| Lin et al. [44]<br><br>USA<br><br>2015 | Colon Cancer (stage III) | National Cancer Data Base<br><br>Patients aged 18 – 80 who had a colectomy within 3 months of diagnosis and survived > 6months<br><br>2007 – 2010<br><br>34,694 | Receipt of adjuvant chemotherapy within 90 days of a colectomy. | Road Distance<br><br>Calculated using Google Maps.<br><br>Distance was treated as a categorical variable using the following categories; 0 – 12.49miles, 12.5-49.9, 50-249, and ≥250miles. For patients flying in from outside the USA for treatment straight-line | Patients' residence at diagnosis (centroid of zip code)<br><br>TO<br><br>Reporting facility (90% had treatment in the reporting facility. | The study found that patients travelling in the further two categories 50 – 249miles and ≥250 miles had a lower likelihood of receiving chemotherapy than those travelling less than 12.5miles. |

| | | | | | | |
|---|---|---|---|---|---|---|
| | | | | distance was used. Average distance travelled to the oncologist was 12.5 miles. | | |
| Maheswaran, et al. [45]<br><br>UK<br><br>2006 | Breast Cancer | Anonymised data<br><br>April 1998 - March 2001<br><br>Sample = 34,868 | Breast Screening Uptake | Road Distance.<br><br>Distance was treated as a categorical variable and a continuous variable. Distances were grouped into 2 km bands. <2km, 2 to <4, 4 to <6, 6 to<8 and ≥8 | Patients' Residence (postcode)<br><br>TO<br><br>The screening location that they were invited to attend. | The study found that when analysed as a continuous variable there was a small but significant decrease in uptake of breast cancer screening with increasing distance - adjusted odds ratio of 0.87 (95% CI -0.79 - 0.95) for a 10km increase in distance. The strongest effect on breast screening uptake was deprivation. |
| Meden, et al. [46]<br><br>USA<br><br>2002 | Breast Cancer | Medical Records.<br><br>1999 – 2000<br><br>Sample = 66 | Difference in treatment technique – Modified Radical Mastectomy vs Breast Conserving Therapy | Distance.<br>Unclear method. Likely to be straight-line.<br><br>Distance was treated as a categorical variable. Distances were split into <45 miles and ≥45miles. Average distance was 61.6 miles (range 0 – 138 miles) | Patients' Residence<br><br>TO<br><br>The nearest radiation oncologist facility. | The study found that access to breast conserving surgery declined as travel distance increased. Patients living further away were more likely to have had a mastectomy. |
| Nattinger, et al. [47]<br><br>USA<br><br>2001 | Breast Cancer | National Cancer Institute - Surveillance, Epidemiology and End Results (SEER) Registry.<br><br>1991 - 1992.<br><br>Sample = 17,729 | Receiving Breast conserving surgery (BCS) OR receiving BCS with radiotherapy. | Straight-line Distance.<br><br>Distance was treated as a categorical variable - using the groups of < 5miles, 5 to <10, 10 to < 15, 15 to < 20, 20 to <30, 30 to <40, ≥ 40 miles for receipt of BCS vs mastectomy and the groups of 0 to <10, 10 to <20, 20 to <30, 30 to <40 and ≥ 40 miles for receipt of radiotherapy among BCS patients. | Patients' Residence (Census tract)<br><br>TO<br><br>The nearest hospital with a radiotherapy facility (centroid of the zip code) | The study found a statistically significant decline in the likelihood of patients undergoing breast conserving surgery living ≥15 miles from a hospital with radiotherapy facilities when compared to those living < 5miles. They also found a statistically significant result for those patients living ≥ 40 miles having a reduced rate of radiotherapy following Breast conserving surgery. |
| Onitilo, et al. [48]<br><br>USA<br><br>2014 | Breast Cancer - Mammography Screening | Local Cancer Registry.<br><br>2002 - 2008.<br><br>Sample = 1,421 | Stage at diagnosis | Road Distance and Travel Time.<br><br>Calculated using ESRI ArcGIS.<br><br>Distances were treated as continuous & categorical variables<br><br>Travel times were split into the categories of 0 - 5 mins, 5 - 15 mins, 15 - 30 mins, 30 - 60 mins, ≥ 60 mins. | Patients' Residence (street address for the patients (where available) /centroid of patients zip code where not)<br><br>TO<br><br>The nearest mammogram facility and the actual facility attended. | The study found that women who missed none of their 5 annual mammograms lived a median of 15 minutes from the nearest facility, whilst those who missed 5 /5 lived a median time of 27 minutes.<br><br>The study found that patients living >30 miles to the closest facility were less likely to be screened for breast cancer in the winter months. |

| | | | | | | |
|---|---|---|---|---|---|---|
| Panagopoulou, et al. [49]<br><br>GREECE<br><br>2012 | Breast Cancer | Hellenic Cooperative Oncology Group (clinical trials in 6 Greek cities)<br><br>1997 - 2005<br><br>Sample = 2,789 (women) | Survival | Road Distance and Travel Time.<br><br>Distance was grouped into < 300km and ≥ 300km. Travel time was grouped into < 4 hours and 4+ hours.<br>Additional tests using the following distance categories: <50, 50 - 149, 150 - 249, 250 - 349, 350+km. | Patients' Residence (98.7% of the sample using residential address, or the city centre of the city of residence, for the remaining 1.3% the weighted mean of available distances to each destination hospital)<br><br>TO<br><br>The treating hospital | The study found that travelling a distance >300km and travel time of 4 + hours were significantly associated with worse survival outcomes (HR = 1.37 & 1.34) base <300km and <4h respectively. |
| Punglia, et al. [50]<br><br>USA<br><br>2006 | Breast Cancer | The linked Surveillance, Epidemiology and End Results- Medicare (SEER) database.<br><br>1991 - 1999.<br><br>Sample = 19,787 | Receiving Radiation Treatment after a Mastectomy | Straight-line Distance.<br><br>Distance was treated as a continuous and categorical variable. Using categories of <25, 25-50, 50-75 and 75+ miles.<br>5 patients living more than 900 miles away were excluded, as were patients in Hawaii.<br><br>The median distance was 4.83 miles. | Patients' Residence<br><br>TO<br><br>The nearest radiation treatment facility. | The study found that increasing distance to the nearest radiation treatment facility was associated with a decreased likelihood of receiving radiation treatment therapy. For each extra 25 miles of travel was associated with declining odds of receiving radiation. The effect of distance showed as being stronger where patients were >75 years and those travelling 75+ miles compared to <25 miles. |
| Schroen, et al. [51]<br><br>USA<br><br>2005 | Breast Cancer | Virginia Cancer Registry. Patients diagnosed<br><br>1996 - 2000.<br><br>Sample = 20,094 | Mastectomy rates VS Breast conservation and radiation therapy | Straight-line Distance.<br><br>Distance was modelled as a categorical using 10 miles, 10 - 25, > 25 - 50 and > 50 miles (range 0 - 84miles) | Patients' Residence ( zip code)<br><br>TO<br><br>The nearest radiation therapy facility. | The study found a higher rate of mastectomy the further distance the patient lived from the nearest radiation therapy facility (after controlling for tumour size, year of diagnosis and age). |
| Scoggins et al. [52]<br><br>USA<br><br>2012 | Breast cancer<br><br>Lung cancer<br><br>Colorectal cancer | Washington State Cancer Registry<br><br>Washing state Medicaid enrolled at time of diagnosis or within 6 months | Stage at diagnosis (local or regional/distant<br><br>Likelihood of surgical treatment. Time to first surgical | Driving Time and Driving Distance<br><br>Calculated using MapQuest (www.mapquest.com) | Patients' residence (9 digit zip code used where available)<br><br>TO | The study found that later stage diagnosis for breast cancer was associated with increased driving time (but not lung or colorectal cancer). There were no significant effects between travel time and likelihood of surgical treatment. A significant result was found for the time to first treatment for colorectal patients where after controlling for socio demographic factors, year of diagnosis, and cancer stage for every 1 hour increase in drive time, time |

| | | | | | | |
|---|---|---|---|---|---|---|
| | | 1997 – 2003<br><br>4,413 | treatment (number of days since diagnosis) | Distance and travel time were treated as categorical variables. The distance categories were: | Patients general practice/ primary care provider | to treatment was delayed by 5.9 days.<br>The study concluded that there was no evidence that drive time was a better predictor than driving distance. |
| Temkin et al. [53]<br><br>USA<br><br>2015 | Gynaecologic cancer | University of Maryland Medical Centre (single site)<br>Nov 2009 – Dec 2011<br><br>152 | Completion of recommended adjuvant therapy | Travel Time and Distance<br>Calculated using the Google Maps.<br>Treated as continuous variables.<br>Distance range 0.3 – 12 miles. Travel time range 2 – 169 mins. | Patients' Residence (zip code)<br>TO<br>The hospital attended | The study found mixed results - 87% of the sample completed the therapy. 11 people did not complete and 8 died before completion. They found that those patients living <10 miles or >50 miles were less likely to complete treatment (13% of the sample). Those living further were more likely to die before completing, but also had higher comorbidities. |
| Thomas et al. [54]<br>Ireland<br>2015 | Colorectal Cancer | Irish National Cancer Registry<br>Patients who were diagnosed and still alive.<br>Oct 2007 – Sept 2009<br><br>1273 sent questionnaires, 496 returned | Quality of life following survival (measured using QLQ-30) | Distance<br>Unspecified method<br><br>Distance was treated as a categorical variable. Distances were divided into tertiles. Groups 1 and 2 were combined (≤30.81km) & group3 (>30.81km). Group 3 was then defined as living "remotely" from the hospital. | Patients Residence (at time of diagnosis)<br>TO<br>The hospital they were treated at. | The study assessed the impact of distance on the components to the QLQ-30. This was then split by gender. The study found that living a greater distance from the hospital was associated with – lower physical functioning and role functioning ( for women and not men). For men living remotely (>30.8km) had a significant negative impact on their overall self-reported health and quality of life, but not for women. |
| Tracey et al. [55]<br><br>Australia<br><br>2015 | Lung Cancer | New South Wales Central Cancer Registry<br>2000 - 2008<br><br>11,457 (split into diagnosis – localised stage, regional and distance) | Survival<br><br>(at one and five years) | Straight-line Distance<br>Calculated using the 'Great Circle distance calculator'<br><br>Distance was treated as a categorical variable using 3 groups of 0-39km, 40-99km and 100+ km. | Patients' Residence<br><br>TO<br><br>The nearest specialist public hospital (NASH) & nearest general hospital. | The study found that patients living further away from the specialist hospitals were less likely to attend the specialist hospital & less likely to have curative surgery – Resulting in lower survival rates. Patients who lived further away & were admitted to a specialist hospital and received curative surgery were more likely to survive at 5 years than those not receiving curative surgery. |
| Tracey et al. [56]<br>Australia<br>2015 | Lung Cancer (localised non-small cell) | NSW Central Cancer Registry<br>Patients admitted with localised stage at diagnosis ≤12 months following diagnosis | Receiving Surgical resection within 12 months of diagnosis | Straight-line Distance<br>Calculated using the 'Great Circle distance calculator'<br><br>Distance was treated as a categorical variable using 3 | Patients' Residence<br><br>TO<br><br>The nearest specialist public | The study found that 51% of patients living >100km from a specialist hospitals didn't have surgery compared to 38% of those living <40km. Patients living further from the specialist hospitals were more likely to be treated at a general hospital and less likely to receive potentially curative surgery. |

| | | | | | | |
|---|---|---|---|---|---|---|
| | | 2000-2008<br>3,240 | | groups of 0-39km, 40-99km and 100+ km. | hospital (NASH) & closest general hospital. | |
| Tracey, et al. [57]<br>AUSTRALIA<br>2014 | Epithelial Ovarian Cancer | New South Wales Cancer Registry.<br>2000 - 2008.<br>Sample = 3411 | Survival | Straight-Line Distance.<br>Distance was treated as a continuous variable and categorical variable for which it was grouped into equal quartiles - 0 - 5km 5.1-9.0km, 9.1-27.0, 27.1 - 187.0, 187.1+ | Patients' Residence<br>TO<br>The closest gynaecological oncology Hospital | The study concluded that there was an increasing trend in the unadjusted hazard of death model with increase in distance to the closest public gynaecological Oncology hospital. The study reported that whilst they had used the closest hospital in their calculations only 37% of their sample had used their closest hospital. |
| Wang, et al. [58]<br>USA<br>2008 | Breast Cancer | Illinois Cancer Registry<br>1998 - 2000<br>Sample = 30,511 (9,077 were classed as late stage) | Late stage diagnosis | Straight-line Distance and Travel Time.<br>Travel times were calculated using the ArcInfo network analysis module – using the minimum road distance when taking account of travel speed. | Patients' Residence (Population weight centroid of zip codes)<br>TO<br>The closest mammography facility & the closest GP. | The study found that travel time to mammography services had no statistically significant association with late stage risk.<br>The study did find that as travel time to the nearest GP increased patients were more likely to have a later stage diagnosis. |
| **Kidney studies** | | | | | | |
| Bello, et al. [59]<br>CANADA<br>2012 | Diabetes & Chronic Kidney Disease (jointly) | Alberta Kidney Disease Network & Provincial Health Ministry<br>2005 - 2009<br>Sample = 31,377 | All-cause mortality, all cause hospitalisation, renal outcomes, ESRD initiation, progression to Egfr< 10mL/min/1.73m) | Road Distance.<br>Distance was treated as a categorical variable. Using the following 6 categories 0-50, 50.1 - 100, 100.1 - 200 and >200km | Patients' Residence ( 6 digit postal code)<br>TO<br>The nearest nephrologist | The study found that when using a base of <50km, patients living >50km were less likely to visit a nephrologist, less likely to have follow up measurements of A1c and urinary albumin within a year. Plus have a higher change of all cause hospitalisation and all-cause mortality. |
| Bello, et al. [60]<br>CANADA<br>2013 | Patients with proteinuria (Kidney Damage) | Alberta Health and Wellness, Alberta Blue Cross, the Northern and Southern Alberta Renal Program and the provincial laboratories of Alberta.<br>2002 - 2009<br>Sample = 1,359,330 | A range of health outcomes.<br>ACEI/ARB use in ≥ 65 year olds, Statin use in ≥ 65 year olds, Timely Referral, All cause mortality, myocardial | Shortest Road distance.<br>Distances were treated as a categorical variable using the groups : 0-50, 501 - 100, 100.1 - 200 and >200km. | Patients Residence (6 digit home postal code)<br>TO<br>The nearest nephrologist. | The study found a statistically significantly higher incidence of stroke and hospitalisations in those travelling a greater distance, but no association for the other outcome measures |

| | | | | | | |
|---|---|---|---|---|---|---|
| | | | infarction, stroke, heart failure, doubling of SCr (Serum creatinine ratio), ESRD (end stage renal disease) and hospitalisations | | | |
| Cho, et al. [61]<br><br>AUSTRALIA<br><br>2012 | Peritonitis<br><br>(Kidney) | ANZDATA Registry<br><br>2003 - 2008<br><br>Sample = 6,610 | A range including - Peritonitis Free - Survival, first peritonitis episode, staphylococcus aureus peritonitis. | Road Distance.<br><br>Calculated using Google Maps.<br><br>Distance was treated as a categorical variable using the groupings - < 100km and ≥100km. The cut off was decided a priori as this is the minimum distance states provide patient assisted transport subsidy schemes to facilitate improved access. | Patients' Residence<br><br>TO<br><br>The nearest peritoneal dialysis unit. | The study found that living ≥100 km away from the nearest peritoneal dialysis unit was not significantly associated with time to first peritonitis episode. The study did find an association between living ≥ 100km away from the nearest unit and increased risk of Staphylococcus aureus peritonitis. |
| Judge, et al. [62]<br><br>UK<br><br>2012 | Renal Replacement Therapy (RRT) - Kidney | UK Renal Registry (UKRR)<br><br>2007<br><br>Incident population = 4607<br>Prevalent population = 36,775 | Renal Replacement Therapy Incidence and Prevalence | Travel Time.<br><br>Average speeds were assigned to roads and GIS transportation software Base Trans CAD used to estimate the minimum travel time.<br><br>Travel time was treated as a continuous and categorical variable split into 4 groups: < 15mins, 15 - 29mins , 29 – 45, & 45+ mins | Patients' Residence (Centroid of the CAS Ward (average 2670 people in each ward))<br><br>TO<br><br>The nearest Dialysis Unit | The study found that patients living >45 min travel time from the nearest dialysis unit were 20% less likely to commence or receive renal replacement therapy than those living < 15 min. |
| Miller, et al. [63]<br><br>CANADA<br><br>2014 | Chronic Kidney Disease | Canadian Organ Replacement Registry (CORR)<br><br>2000 - 2009 | Incident Central Venous Catheter (CVC) use | Straight-line Distance.<br><br>Distances were divided into 3 groups <5km, 5 - 20km and >20km | Patients' Residence ( home postal code at dialysis initiation<br><br>TO | The study found that increasing distance was associated with increased use of central venous catheters in incident dialysis patients. |

| | | | | | | |
|---|---|---|---|---|---|---|
| | | Sample = 26,449 | | | The nearest dialysis centre | |
| Moist, et al. [64]<br><br>USA<br><br>2008 | Kidney Dialysis | Dialysis Outcomes and Practice Patterns Study (DOPPS) - questionnaire<br><br>1996 - 2001 (DOPPS 1) 2002 - 2004 (DOPPS 2)<br><br>Sample = 20,994 (from 7 countries, France, Germany, Italy, Japan, Spain, UK and USA) | HRQOL (Health Related Quality of Life), Mortality, Adherence, withdrawal, hospitalisation and transplantation | Travel Time.<br><br>The study was based on a survey which asked the question - How long does it take you to get to your dialysis unit or centre (1 way)? Respondents could answer ≤15mins, 16 - 30, 31 - 60 and >60mins. They were also asked how they usually travelled to the dialysis unit. | Patients' Residence<br><br>TO<br><br>The dialysis centre attended | The study found that longer travel times were associated with a greater adjusted relative risk of mortality. Health related quality of life scores were lower for those with longer travel times when compared with travelling < 15mins. |
| Thompson, et al. [65]<br><br>USA<br><br>2012 | Kidney Disease | United States Renal Data System.<br><br>Jan 1995 – 2007<br><br>Sample = 726,347<br><br>(the study excluded patients with missing or invalid postcodes) | Mortality | Shortest Driving Distance.<br><br>Distance was treated as a categorical variable.<br><br>Using 5 categories: 0-10 miles, 11-15, 26-45, 46-100 and >100miles. The categories correspond to the 0 – 75$^{th}$ , 75-95$^{th}$, 95$^{th}$ -99$^{th}$, 99$^{th}$-99.9$^{th}$ and >99.9$^{th}$ percentiles. | Patients' Residence ( 5 digit zip code at time of first renal replacement, dialysis or transplant)<br><br>TO<br><br>The closest Haemodialysis Centre | The study found that distance, but not living in a rural area was associated with increased mortality. The adjusted model identified a statistically significant hazard ratio between the reference case (0-10milles) and the 11-25 miles and >100miles categories, but not for other distance categories. |
| Thompson, et al. [66]<br><br>USA<br><br>2013 | Kidney | United States Renal Data System<br><br>2001 - 2010<br><br>Sample = 1,784 | Quality of Care Indicators (90 days following haemodialysis therapy and at 1 year) | Shortest Road Distance.<br><br>Distance was treated as a categorical variable. Using the following categories: ≤50km, 50.1 - 150km, 150.1 - 300, >300km. | Patients' Residence ( 5 digit zip code)<br><br>TO<br><br>The closest nephrologist. | The study found that patients were less likely to have seen a Nephrologist 90 days prior to starting haemodialysis therapy, and were more likely to have a sub optimal levels of phosphate control the further they lived from a haemodialysis centre. |
| Tonelli, et al. [67]<br><br>CANADA<br><br>2007 | Kidney Failure | Canadian Organ Replacement registry<br><br>1990 - 2000<br><br>Sample = 26,775 | Mortality | Shortest Road Distance<br><br>Calculated using postal data converted using www.melissadata.com and entered into ArcGIS.<br><br>Distance was treated as a categorical variable using the | Patients' Residence (6 digit postal code)<br><br>TO<br><br>The practice location of the patients' nephrologist. | The study found that remote dwelling Canadians with kidney failure were significantly more likely to start renal replacement on Peritoneal Dialysis (PD) and switch to PD if their initial dialytic option was haemodialysis. The adjusted rates of death and the adjusted hazard ratios were significantly higher in those living ≥50km from the nephrologist compared to those < 50 km. |

| | | | | | | |
|---|---|---|---|---|---|---|
| | | | | groups of: <50km, 50.1 - 150km, 150.1 - 300 and >300km | | |
| Tonelli, et al. [68]<br><br>Canada<br><br>2007 | Kidney (Haemodialysis) | Canadian Organ Replacement Register<br><br>1990 - 2000 (when the sample started dialysis)<br><br>Sample = 18,722 (random sample of 75% of the patient population) | Mortality (from all causes) Then split by cause - infectious or cardiovascular | Shortest Road Distance<br><br>Calculated using ArcGIS 9.1.<br><br>Distance was treated as a categorical variable using the following groups - 0-50km, 50.1-150km, 150.1-300km, >300km | Patients' Residence<br><br>TO<br><br>The practice location of the attending nephrologist. | The study found that mortality associated with haemodialysis was greater for patients living further from their attending nephrologist. This was particularly evident for infectious causes. |
| Diabetes Studies | | | | | | |
| Littenberg, et al. [69]<br><br>USA<br><br>2006 | Type 2 diabetes | Vermont Diabetes Information System. Adults completed postal surveys and were interviewed at home.<br><br>Years Unknown<br><br>Sample = 781 (131 insulin users & 650 non users) | Glycaemic Control Insulin Use | Shortest driving distance<br><br>Calculated using ESRI ArcView 3.3 and a geographic data set of roads from Tele Atlas.<br><br>Distance was treated as a continuous and categorical variable. Distances were grouped as <10km & > 10 km | Patients' Residence (home address)<br><br>TO<br><br>Primary care facility | The study found that insulin users had shorter driving distances to the healthcare facility than non-users. Longer driving distances were associated with poorer glycaemic control. The OR for those using insulin, living <10km, having glycaemic control was 2.29 (CI 1.35, 3,88; p = 0.002). |
| Strauss, et al. [70]<br><br>USA<br><br>2006<br><br>(Data cross over with Littenberg et al 2006)) | Diabetes | Vermont Diabetes Information system. Adults completed postal surveys and were interviewed at home<br>(23% of the contacted population)<br>July 2003 - March 2005<br><br>Sample = 973 (794 non insulin users & 179 insulin users) | Glycaemic Control (for insulin and non-insulin users) | Shortest Road Distance<br><br>Calculated using a road network in ArcvIEW 3.3.<br><br>Distance was modelled as a categorical variable. Patients were split into 3 equal groups <3.8km, 3.9 - 13.3km, ≥13.3km | Patients' Residence ( home address)<br><br>TO<br><br>Primary care facility used. | The study identified that longer driving distances from the patients' home to the site of primary care were associated with poorer glycaemic control. |
| Zgibor, et al. [71]<br><br>USA | Diabetes | Seven diabetes management centres in Southwestern | Controlled vs uncontrolled diabetes | Road Distance.<br><br>Driving distance using the | Patients' Residence ( home address) | The study found that living > 10 miles away significantly contributed to lower levels of glycaemic control for diabetes patients. Those who lived ≤ 10 |

| | | | | | | |
|---|---|---|---|---|---|---|
| 2011 | | Pennsylvania.<br><br>Jun 2005 - Jan 2007<br><br>Sample = 3,369 | | network analyst tool in ArcGIS. Distance was treated as a continuous and categorical variable.<br>Distance was divided into 2 categories ≤10 miles and >10 miles.<br>The average distance was 13.3 miles. | TO<br><br>The diabetes treatment centre attended. | miles from the diabetes treatment facility were 2.5 times more likely to have improved their levels of glycaemic control between their first and last visits. |
| Transplant Studies | | | | | | |
| Goldberg, et al. [72]<br><br>USA<br><br>2014 | Liver Transplant | Veterans Health administrations integrated, national electronic medical records linked to organ procurement and transplantation network<br><br>2003 - 2010<br><br>Sample = 50,637 | Being waitlisted for a liver transplant, having a liver transplant and mortality | Straight-line Distance.<br><br>Distance was treated as both a continuous and categorical variable.<br><br>5 distance categories: 0 - 100miles, 101-200, 201-300, 301-500, >500miles | Veterans Admission (VA) Centre<br><br>TO<br><br>The Veterans Admission Transplant Centre (VATC) | The greater the distance from a VATC or any transplant centre was associated with a lower likelihood of being put on a waiting list or receiving a transplant and greater likelihood of death. |
| Redhage, et al. [73]<br><br>USA<br><br>2013 | Liver Transplant | Hospital Data and HRQOL (Health Related Quality of Life) survey.<br><br>Dates unknown<br><br>Sample = 706 | Longitudinal HRQOL was measured using the SF-36 Health Survey and a rolling enrolment process. | Distance<br><br>[unspecified]<br><br>Distance treated as a continuous variable. The distance range was 0 – 2261 miles and average 179. | Patients' Residence (home address )<br><br>TO<br><br>The transplant centre | The study found that increased distance to the transplant centre was associated with a decreased post-transplant physical HRQOL, but that there was no association between distance and pre- transplant HRQOL. |
| Thabut, et al. [74]<br>USA<br><br>2012 | Lung Transplant | Transplant Registry<br><br>2001- 2009<br><br>Sample = 14,015 | Listing for a transplant, receipt of a transplant and survival. | Straight-line Distance.<br><br>Using ArcGIS Software.<br>Distance was treated as a categorical variable using two different sets of groupings.<br><br>Firstly - the following groups - 0 - 50 miles, 51 - 100 miles, 101 - 150 miles, 151 - 200 miles and > 200 miles. Secondly - 6 | Patients' Residence (centroid of the residential zip code)<br><br>TO<br><br>The nearest adult lung transplant centre | The study found that the distance from a lung transplant centre was inversely associated with the hazard of being listed (both before and after the introduction of the lung allocation score). Once waitlisted distance from the closest centre was not associated with differences in survival. |

| | | | | | | |
|---|---|---|---|---|---|---|
| | | | | categories 0 - 50th percentile, 50th - 75th percentile, 75th - 90th percentile, 90th to 95th percentile, 95th - 99th percentile and + 99th. | | |
| Zorzi, et al. [75]<br><br>USA<br><br>2012 | Liver Transplant | United Network for Organ Sharing<br><br>Jan 2004 – July 2010<br><br>Sample = 5,673 | Mortality & being dropped from a waiting list due to being too sick. | Straight-line Distance.<br><br>Distance were calculated using ww.zip-codes.com<br><br>Distance was considered as a continuous & categorical variable and divided into the following 3 groups: <30miles, 30 -60 miles and >60 miles | Patients' Residence<br><br>TO<br><br>The nearest liver specialised transplant centre & nearest 300 bed hospital. | The study found that increased distance from a specialised liver transplant centre was associated with an increased likelihood of death. The likelihood of wait list drop out was significantly higher for patients living > 30 miles from the specialised liver transplant centre. |
| Obesity Studies | | | | | | |
| Jennings, et al. [76]<br><br>UK<br><br>2013 | Obesity (Laparoscopic adjustable gastric banding - LAGB) | Hospital Database.<br><br>< 2010.<br><br>Sample = 227 | Compliance with follow up appointments. | Road Distance.<br><br>Calculated using Google Maps.<br><br>Distance was treated as a continuous variable. The average distance for perfect attenders is 15.3 miles and non-attendees are 21.1.miles. | Patients' Residence (Home Address)<br><br>TO<br><br>The treating hospital | The study identified that compliance with follow up following LAGB is associated with better weight loss. Patients living closer to the treating hospital were more likely to regularly attend follow up. The study reported longer public transport journey times in the non-attending group - but did not include this in the analysis. |
| Lara, et al. [77]<br><br>USA<br><br>2005 | Obesity | Gundersen Lutheran Medical Centre data.<br><br>Sept 2001 - April 2003<br><br>Sample = 150 | Compliance with follow up at 3, 6 ,9 and 12 month appointments | Straight-line Distance.<br><br>Distances were treated as a categorical variable using groups: <50 miles 50 - 100 miles and >100 miles | Patients' Residence ( zip code<br><br>TO<br><br>The Clinic they were treated/ followed up at. | The study found that travel distance from the clinic did not significantly affect compliance at the initial follow-up, 3-month, and 12-month appointments. However, distance did affect compliance at the 6-month appointment and significantly affected compliance at the 9-month appointment. |
| Sivagnanam and Rhodes [78]<br><br>UK<br><br>2010 | Obesity - Laparoscopic adjustable gastric band (LAGB) | The Norwich Spire Hospital.<br><br>October 1997 - March 2009.<br><br>Sample = 150 | Follow up and weight loss | Distance.<br><br>Method not reported.<br><br>Distance was treated as a categorical variable and split into the following distance groups <10, 10 - 20, 20 - 30 and > 30. (all miles) | Patients' Residence<br><br>TO<br><br>The Norwich Spire Hospital. | The study found that patients attended fewer follow up clinics, as distance increased from the patient's home address. The percentage estimated weight loss was lowest in the group that lived furthest from the hospital, but this was not statistically significant. |

| | | | | | | |
|---|---|---|---|---|---|---|
| | | | | 87% of the patients lived < 50 miles from the hospital. | | |
| Mental Health Studies | | | | | | |
| McCarthy, et al. [79]<br><br>USA<br><br>2007 | Mental Health - Schizophrenia or bipolar disorder | National Veterans Affairs (VA) administrative data. Patients who received a diagnosis of schizophrenia or bipolar disorder in the year<br><br>Oct 1997 - Sept 2008 and survived the year.<br><br>Sample = 163,656 | Continuity - measured by time to first 12 month gap in VA health services utilisation | Straight-line Distance.<br><br>Distance was treated as a continuous variable. Average distance to the nearest provider was 11.8 miles. | Patients' Residence (population centroid of the patients zip code)<br><br>TO<br><br>The nearest VA providers of substantial psychiatric services or community based outpatient clinics serving at least 500 unique patients where at least 20% were mental health visits. | The study found that patients who had a 12 month gap in VA services utilisation were more likely to have a lower Charlson comorbidity score and live further away.<br><br>Living ≥25 miles from VA care was associated with a greater likelihood of a gap in VA health utilisation. The hazard ratio associated with each 5 miles further from psychiatric services was 1.011. |
| Joseph and Boeckh [80]<br><br>CANADA<br><br>1981 | Mental Health | Provincial health records<br><br>1976<br><br>Sample = 1767 inpatients & 883 outpatients | Seriousness of diagnosis | Distance.<br><br>Distance from Peterborough Ontario. They do not provide any other information on method of calculation. | Patients' Residence<br><br>TO<br><br>Peterborough Ontario | The study concluded that severity of diagnosis increased as distance travelled increased. |
| Skarsvag and Wynn [81]<br><br>NORWAY<br><br>2004 | Mental Health Psychiatric | Regional population & actual patient data from the Stokmarknes Clinic in Nordland<br><br>1992 - 1996<br><br>Sample = 10,996 (total population)<br><br>Sample = 1,834 treated population. | Use of an outpatient clinic | Travel Time.<br><br>Calculated from information gathered from local bus and ferry companies.<br><br>The study treated travel time as a categorical variable using the cut off of 35 minutes. | All residential addresses in the local area & actual patient attendees.<br><br>TO<br><br>The outpatient clinic at Stokmarknes. | The study found that a significantly higher proportion of those living < 35 mins from the clinic had used the clinics services than > 35mins. |
| **Other studies** | | | | | | |
| Allen et al. [82]<br><br>USA | Sleep Apnea | University of British Columbia Hospital Sleep Disorders Clinic<br><br>Included referred | Severity of obstructive sleep apnea | Travel Time.<br><br>Calculated using DMTI routing data and the ArcGIS Network analyst function. | Patients' Residence (postcode)<br><br>TO<br><br>The sleep disorder clinic | The study found that travel time to the sleep clinic was a predictor of obstructive sleep apnea severity (controlling for sex, age, obesity and education). Every 10 min increase in travel time was associated with an increase of 1.4 events per hour in the apnea-hypopnea index. |

| | | | | | | |
|---|---|---|---|---|---|---|
| 2016 | | patients whose travel times were < 1 hour.<br>May 2003 – July 2011.<br>1,275 | | Travel time was treated as a continuous variable and categorical variable. The mean travel time was 20.8 mins. The cut point for the categorical variable was the mean time. | | |
| Arcury, et al. [83]<br>USA<br>2005 | Non specific - Health care visits | Survey of adults in 12 rural Appalachian North Carolina Counties. Personal interviews in participants homes.<br>1999 - 2000.<br>Sample = 1,059 | Number of regular check-up care visits, chronic care visits and acute care visits | Straight-line Distance.<br>Distance to the healthcare facility was based on respondents stating which hospital, clinic or doctor they would normally go to for "a really bad emergency", A less serious emergency, and for regular care. The average distance for regular check-up visits was 14 miles, for chronic care visits 18 miles and serious emergencies 18.58miles. | Patients' Residence (Survey at respondents homes )<br>TO<br>The self-reported hospital, GP, clinic that they would normally go to for a really bad emergency, a less serious emergency or for regular care. | The study found that distance was significantly associated with the number of regular check-up care visits and chronic care visits. Distance was not associated with acute care visits.<br>They identified that those people with a driving license had an estimated 1.58 times more regular care visits and 2.3 times more chronic care visits. |
| Ballard, et al. [84]<br>USA<br>1994 | Non-specific. | Medicare hospitalization data (MEDPAR)<br>1998<br>Sample = 13,596 Two groups – patients referred to Mayo Rochester hospitals and separately national referral hospitals. | 30 day mortality | Distance<br>No information in paper on specific method.<br>Distance was split into the categories of <10 miles and ≥ 10 miles. | Patients' Residence ( zip code)<br>TO<br>The hospital attended (zip code) | The study found that increased distance from the patient's residence to the hospital that they were treated in was independently associated with higher 30 day mortality rates. |
| Chou, et al. [85]<br>USA<br>2012 | Coronary Atery Bypass Graft (CABG) | Pennsylvania HealthCare Cost Containment Council<br>1995 - 2005<br>Sample = 102,858 | In hospital mortality and readmission | Straight-line distance.<br>Distance was treated as a continuous variable.<br>Average distance 14.9 miles. | Patients' Residence (Centroid of the patient's residential zip code)<br>TO<br>The admitting hospital | The study found that high risk CABG patients living further from the admitting hospital had increased in-hospital mortality. |
| Etzioni, et al. [86]<br>USA | Any Surgical Operation | National Surgical Quality Improvement Project (NSQIP) | 30 day surgical outcomes | Distance<br>No information on method. | Patients' Residence ( zip code centroid) | The study found that patients who lived closer were less likely to have a serious complication at 30 days and had better outcomes than predicted. |

| | | | | | | |
|---|---|---|---|---|---|---|
| 2013 | | database - for a large tertiary care institution.<br><br>2006 - 2009<br><br>Sample = 6,938 procedures | | Distances were treated as a categorical variable and split into quintiles by procedure category. This allowed the study to take into account that patients travelled further for more complicated operations. The average distance was 226 miles. | TO<br><br>The tertiary hospital attended. | |
| Evans et al. [87]<br><br>USA<br><br>2016 | HIV with Severe sepsis | University of Virginia Clinical data repository<br><br>2001 – [not stated]<br><br>74 | In hospital Mortality | Distance<br><br>Method unspecified.<br><br>Dichotomised into ≤40miles and >40 miles | Patients' Residence (assumed)<br><br>TO<br>The University of Virginia Ryan White HIV clinic | The study found that after adjusting for severity of illness and respiratory failure, patients living >40 miles from the clinic had a fourfold increased risk of in-hospital mortality compared to ≤40 miles. |
| Haynes, et al. [88]<br><br>UK<br><br>1999 | Inpatient Episodes | Regional Health Authority.<br><br>1991 - 1993<br><br>Sample = 470,650 acute episodes, 13,425 psychiatric episodes and 36,909 geriatric episodes. | Healthcare episodes | Straight-line Distance.<br><br>Distance was treated as a continuous variable. The furthest distance to the GP was 8km and to the acute hospitals 41km. | Patients' Residence (population weighted centroid of the patients ward)<br>TO<br>The nearest district general hospital.<br><br>&<br><br>Patients' Residence<br>TO<br>The nearest GP surgery. | The study found that after controlling for key confounders distance to hospital was a significant predictor of hospital episodes, especially psychiatric episodes.<br><br>The study found that distance to the GP was only significantly associated with reductions in acute episodes in hospital. |
| Jackson, et al. [7]<br><br>USA<br><br>2013 | Colorectal Surgery | The National Surgical Quality Improvement Programme Database.<br><br>May 2003 - April 2011<br><br>Sample = 866 | Length of Stay | Road Distance with the shortest travel time.<br><br>Distance was treated as a continuous variable. The mean distance travelled was 146.9 miles (range 2 - 2984). The study transformed distance and length of stay onto the log scale due to non-normal distributions. | Patients' Residence (5 digit zip code)<br><br>TO<br><br>The hospital treated at (5 digit zip code). | The study found that in the adjusted model increased travel distance from a patient's residence to the hospital was associated with an increase in length of stay. |
| Jackson, et al. [89]<br><br>USA | Elective Pancreatic Surgery | Local National Surgery Quality Improvement database. | Length of Stay | Road Distance (shortest travel time) | Patients' Residence (5 digit zip code) | The study found (in the general model) that for each additional 100 miles travelled, the length of hospital stay increased by 2%. |

| | | | | | | |
|---|---|---|---|---|---|---|
| 2014 | | 2005 - 2011<br><br>Sample = 243 | | Distance was treated as a continuous variable. The distances ranged from 3 - 3006 miles. | TO<br><br>The hospital treated at (5 digit zip code) | |
| Jones, et al. [90]<br><br>UK<br><br>1999 | Asthma | Regional Deaths System for East Anglia.<br><br>1985 - 1995<br><br>Sample = 768 (of which asthma was the underlying cause of death in 365 of these). | Mortality | Travel Times.<br><br>Travel times were treated as categorical & continuous variables.<br><br>The groupings used for travel to the GP were 0 - 4mins >4 - 6 mins, >6 - 9 mins and ≥ 9mins. The minimum travel time was 3 minutes and the maximum 20.8 minutes.<br>The groupings used for travel time to the hospital were 0 - 10, > 10 - 20, > 20-30, ≥ 30mins. The minimum time to the hospital was 4.4 minutes and the maximum 54.7 minutes. | Patients Residence (starting point measured at the ward level-average number of households = 2,726)<br><br>TO<br><br>The nearest GP and the nearest acute hospital with over 200 beds. | The study identified an association between asthma mortality and increasing travel time to the nearest acute hospital. The study found no relationship between distance to the GP and asthma mortality rates. |
| Lake, et al. [91]<br><br>UK<br><br>2011 | TB - treatment with full course of anti TB therapy | National enhanced TB surveillance system (ETS)<br><br>2001 - 2006<br><br>Sample = 21,954 | Completion of TB Treatment | Road Distance.<br><br>Distance was treated as a categorical variable using the groups of < 7.3km and > 7.3km. | Patients' Residence (postcode)<br><br>TO<br><br>The TB treatment facility | The results indicate that attending a TB centre with low case load or greater distance was associated with poorer treatment outcomes. The study identified that distance to a TB treatment centre was insignificant for patients native to the country (UK). |
| Lankila et al. [92]<br><br>Finland<br><br>2016 | Primary Healthcare Attendance | Northern Finland 1966 Birth Cohort<br><br>Questionnaire administered 1997 (cohort were all 31 years old)<br><br>4,503 | Use of local health centres | Shortest Road Distance<br><br>Calculated using the Finish road network data (Digiroad) using ESRI ArcGIS 10.<br><br>Distance was treated as a categorical variable using 0-1.9km, 2 – 4.9 km 5.0-9.9 km and ≥10.0km | Patients' Residence<br><br>TO<br><br>The municipalities health centre facility (or where there were more than one – the closest was used) | The study found that the number of people attending health centres and mean number of visits declined with distance for people living in rural areas, but this was not significant, but the opposite was the case for the sub group in urban areas travelling ≥10.0km compared to 0-1.9km. |
| Monnet [93] | Hepatitis C | Registry Data<br><br>1994 - 2001 | Hepatitis C detection rates | Road Distance.<br><br>Calculated using Chrono Map in | Patients' Residence (geometric centroid of the patients municipality of | The study found that the detection rate for Hepatitis C decreased in each of the studies socioeconomic clusters as distance to the GP increased. |

| | | | | | | |
|---|---|---|---|---|---|---|
| FRANCE<br><br>2008 | | sample = 1,938 | | MapInfo with the 1997 Michelin light road network table (which includes major roads).<br><br>Distance was treated as a continuous variable. | residence)<br><br>TO<br><br>The GP (geometric centroid of municipality) | |
| Prue, et al. [94]<br><br>USA<br><br>1979 | Alcohol Abuse | Jackson Veterans Administration Hospital.<br><br>Years Unknown,<br><br>Sample = 40. | Aftercare attendance. | Road Distance.<br><br>Calculated as total miles. Split into "miles to " the nearest highway and "miles on" the nearest highway.<br><br>Distance was treated as a continuous variable. The range of distances was (12 - 378 miles). | Patients' Residence ( home address)<br><br>TO<br><br>The aftercare facility | The study found that the number of "miles to" and "miles on" the highway significantly affected the probability of attendance at an alcohol abuse aftercare appointment. Distance to the major highway was more predictive of attendance than the miles on the major highway. |
| Singh, et al. [95]<br><br>CANADA<br><br>2014 | Cardiac | Brunswick Cardiac Centre.<br><br>2004 - 2011.<br><br>Sample = 3,897 | 30 day rates of adverse events following non-emergency cardiac surgery | Road Distance.<br><br>Distance was treated as a categorical variable using the following groupings: 0-50km, 50 - 100km, 100 - 150km, 150 - 200km, 200 - 250km and >250km. | Patients' Residence (Home address)<br><br>TO<br><br>The Cardiac Surgery Centre | The study found that increased distance from the cardiac surgery centre was independently associated with a greater likelihood of experiencing an adverse event at 30 days. |

## Supplement table 3: Included studies identifying evidence of a distance bias association

| Author<br><br>Country | Disease / Procedure | Source,<br>Years &<br>Sample size | Health Outcome | Distance/ travel time measurement | Origin and Destination | Summary of key results |
|---|---|---|---|---|---|---|
| Cancer Studies | | | | | | |
| Bristow et al.[96]<br><br>USA<br><br>2015 | Ovarian Cancer (Advanced Stage) | Californian Cancer Registry<br><br>1996 – 2006<br><br>11,765 | Mortality | Straight-line Distance<br><br>Calculated using ESRI ArcMap 10.0.<br><br>Distance was treated as a categorical variable using quintiles. Categories for hospital attended: <5km, 5-9, 10-16, 17-31, ≥32km. Categories for nearest high volume hospital: <9km, 9-17, 11-20, 21-49 & ≥80km.<br><br>80% of patients travelled ≤28.3km to the hospital they were treated at. 80% of patients were ≤ 79.6km to the nearest high volume hospital. | Patients' Residence<br><br>TO<br><br>The hospital treated at and the nearest high volume hospital. | The study found that travelling 5-9km, 17-31 km and ≥32km to the hospital compared those travelling <5km (reference case) was associated with a reduction in the risk of mortality. After controlling for hospital size and adherence to treatment guidelines 5-9km and 17-31km compared to the reference case were still significant.<br><br>The opposite case was found for distance to the nearest high volume hospital for patients travelling ≥80km compared to the reference case of <9km. This was no longer significant after controlling for adherence to treatment guidelines. |
| Lamont, et al.[97]<br><br>UK<br><br>2003 | Cancer | 4 phase II chemo radiotherapy studies conducted at the University of Chicago.<br><br>1993 - 2000<br><br>Sample = 110. | Survival | Distance.<br><br>Driving miles (using an "internet based mapping engine").<br><br>Distances were treated as a categorical variable and split into two groups ≤ 15 miles (45 patients) and > 15 miles (67 patients) | Patients Residence (exact address)<br><br>TO<br><br>The University of Chicago hospital | The study found a positive association between the distance that patients travelled and survival. Those living > 15 miles had only 1/3 of the hazard of death than those living ≤15 miles. With every 10 miles that a patient travelled the hazard of death declined by 3.2%. |
| Lenhard Jr, et al.[98]<br><br>USA<br><br>1987 | Multiple Myeloma | Centralised Cancer Patient Data System.<br><br>1977 - 1982.<br><br>Sample = 1,479 | Survival | Distance.<br><br>Distance was treated as a categorical variable using the following groups - 0 - 9 miles, 10 - 49 miles, 50 - 149 miles, and ≥ 150miles | Patients' Residence ( zip code)<br><br>TO<br><br>The treating centre (zip code area) | The study found that survival improved with increasing distance travelled to treatment centres. |

| | | | | | | |
|---|---|---|---|---|---|---|
| Lipe, et al. [99]<br><br>USA<br><br>2012 | Bone Marrow Transplant for Multiple Melanoma | Dartmouth Hitchcock Medical Centre transplant registry<br><br>1996 - 2009<br><br>Sample = 77 | Survival (OS and progression free survival) | Straight-line Distance.<br><br>Calculated using www.melissadata.com.<br><br>Distance was treated as a continuous variable and categorical variable split into the groups of < 50miles and > 50 miles | Patients' Residence<br><br>TO<br><br>The Dartmouth Hitchcock Medical Centre | The study found that increasing distance from the transplant centre was associated with improved overall survival. The authors identified that this could be due to a referral bias, but could also be due to a healthier and more motivated groups of patients living further away. |
| Wasif, et al. [100]<br><br>USA<br><br>2104 | Gastrointestinal Cancer | National Cancer Database.<br><br>2003 – 2009<br><br>Sample = 77 | Survival | Distance.<br><br>[Method not specified]<br><br>Distance was treated as a continuous variable and categorical variable split into the groups of <50 miles and >50 miles | Patient' Residence (zip code centroid)<br><br>TO<br><br>The treatment facility zip code centroid | The study found that adjusted hazard ratios were significantly lower for patients travelling > 50 miles compared to < 50 miles. This was true for liver, oesophageal and pancreatic cancer. They concluded that those that travelled > 50 miles to the treatment facility had lower 30 day mortality rates. |
| Other Studies | | | | | | |
| DeNino, et al. [8]<br><br>USA<br><br>2010 | Obesity (Gastric Band) | Teaching hospital patients<br><br>Nov 2008 - Nov 2009<br><br>Sample = 116 | Follow Up Compliance and Weight Loss | Road Distance.<br><br>Calculated using Google Maps.<br>Distance was treated as a continuous variable. The average distance to the hospital was 39.5 miles. | Patients' Residence (exact address)<br><br>TO<br><br>The hospital treated at. | The study found a weak relationship between increased travel distance to the hospital and increased weight loss.<br>Travel distance was found not to be significant for attending follow up visits. |

## Supplement table 4: Included studies identifying no association

| Author<br><br>Country | Disease / Procedure | Source, Years & Sample size | Health Outcome | Distance/ travel time measurement | Origin and Destination | Summary of key results |
|---|---|---|---|---|---|---|
| Cancer Studies | | | | | | |
| Celaya, et al. [101]<br><br>USA<br><br>2010 | Breast Cancer | New Hampshire State Cancer Registry (NHSCR)<br><br>1998 - 2004<br><br>Sample = 5,966 | Stage at diagnosis | Driving Time and Road Distance.<br><br>Calculated using ESRI ArcGIS and data from ESRI on street networks, posted speed limits and driving distance.<br><br>Distance and travel time were treated as categorical variables.<br>Using the following groupings: < 5 miles, 5 - <10 miles, 10 - < 15.0 miles, ≥15 miles.<br>For travel time < 5 mins, 5 - < 10 mins and ≥ 10 mins | Patients' Residence (Addresses of patients were geocoded to an exact street address(91%) or to the zip code centroid if only a post office box or rural route address was available.)<br><br>TO<br><br>The nearest mammography facility. | The study identified no significant association between later stage breast cancer and travel time to the nearest mammography facility. They did identify that there was good access (patients did not have to travel a large distance) to mammography facilities in the area studied, as shown by the categorical groupings. |
| Cosford, et al. [102]<br><br>UK<br><br>1997 | Cancer | Cancer Registry<br><br>1991<br><br>Sample = described as the no. of people in each local authority district attending hospital with a diagnosis of cancer and the no. who received radiotherapy in that year. | Radiotherapy uptake | Travel Time.<br><br>Modelled used to obtain off peak drive times + use of "commercially available computer programme".<br><br>Travel time was treated as a continuous variable.<br><br>Maximum travel times 1 hour. | Population weighted centroid of 14 different local authorities<br><br>TO<br><br>The nearest cancer centre serving the area. | The study found no significant relationship between overall radiotherapy uptake and travel times. |
| Crawford, et al. [103]<br><br>UK<br><br>2012 | Colorectal Cancer | Northern and Yorkshire Cancer Registry and Information Service.<br><br>1994 – 2002<br><br>Sample = 39,619 | Stage of diagnosis & receipt of treatment | Travel Time.<br><br>Shortest road route and average driving speeds along the routes by road class. Travel times were split into quartiles. | Patients' Residence<br><br>TO<br><br>The nearest hospital providing diagnostic and surgical treatment services for bowel cancer. | The study found no effect of travel time distance on stage of diagnosis or receipt of treatment. They also found no interaction effects between deprivation and travel time. |

| | | | | | | |
|---|---|---|---|---|---|---|
| Gunderson, et al. [104]<br><br>USA<br><br>2013 | Cervical Cancer | Medical Records<br><br>2006 - 2011<br><br>Sample = 219 | Overall Survival<br><br>Progression free survival | Straight- line Distance.<br><br>Distance was treated as a categorical variable. Using the following groups: <30 miles and >30 miles | Patients' Residence (zip code)<br><br>TO<br><br>The treating hospital (if the patient underwent surgery) otherwise the radiation centre. | The study found no significant difference between patients travelling <30 miles and those travelling >30 miles for survival. They found that non Caucasians were less likely to travel > 30 miles. |
| Heelan and McKenna [105]<br><br>IRELAND<br><br>2011 | Cancer | Melanoma Database.<br><br>2000 - 2009<br><br>Sample = 106 | Breslow Thickness | Driving Distance.<br><br>The automobile Association route planner was used to estimate distance travelled by road.<br><br>Data was treated as a categorical variable using the groupings of < 30km and >30km. The median distance was 33.3km (range 0.2 - 123.12km) | Patients' Residence<br><br>TO<br><br>The hospital attended. | The study found no significant association between distance travelled and Breslow thickness on presentation. The study concluded that this could have been due to the type of patients in the sample (high number of thick lesions) in both distance categories. |
| Henry, et al. [106]<br><br>USA<br><br>2013 | Breast Cancer | US North American Association of Central Cancer Registries.<br><br>Patients diagnosed 2004 - 2006<br><br>Sample = 174,609 | Stage at diagnosis | Travel Times.<br><br>The study calculated 3 accessibility measures including shortest road network drive time. This used the NAACCR shortest path calculator. - https://www.naaccr.org/Research/ShortestPathFinder.aspx<br><br>Travel times were treated as categorical variable using the following groups - ≤ 5 mins, > 5 - 10, > 10 - 20, > 20 - 30, > 30. 93% of the breast cancer cases lived < 20 mins from the nearest mammography facility and only 2.8 % lived > 30mins. | Road nearest the population weighted centroid of each census tract<br><br>TO<br><br>The nearest FDA certified mammography facility | The study found that after adjusting for poverty there was no impact of distance on late stage diagnosis. They found that poverty was independently associated with late stage diagnosis. |
| Henry, et al. [107]<br><br>USA<br><br>2011 | Breast cancer | 10 state population based cancer registries - covering 30% of the population of the USA. Patients diagnosed<br><br>2004 - 2006<br><br>Sample = 161,619 | Stage at Diagnosis | Travel Time.<br><br>Travel time was modelled as both a continuous and categorical variable. There were 7 categories ranging from < 10 mins to ≥ 60 mins. 76% of the women lived <20 mins from their diagnosing facility & 93% < 20mins from the nearest mammography facility. | Patients' Residence (residential street address (87%) or postal delivery area centroid (8%).<br><br>TO<br><br>The diagnosing facility and nearest facility. | The study concluded that increased travel time was not a determinant of late stage diagnosis. They found that insurance status, race and poverty were associated with risks for a late stage diagnosis of breast cancer. |
| Khera et al.[108] | Hematopoietic cell | Fred Hutchinson Cancer Research Centre/ | Non relapse mortality | Distance | Patients' Residence (zip code) | The study found no relationship between increasing distance and non-relapse mortality, |

| | | | | | | |
|---|---|---|---|---|---|---|
| USA<br><br>2016 | transplantation | Seattle Cancer Care Alliance<br><br>2000 – 2010<br><br>2,849 | Relapse mortality Survival at 200 days | Method unspecified.<br><br>Distance was treated as a continuous and categorical variable. Categories ≤100km, 100- 500, 500, 1000 and > 1000km from the centre were used. Categories of <170km and ≥170 km were used to assess mortality. Median distance 263km (range 0 – 2740km) | TO<br><br>The transplant centre (Fred Hutchinson Cancer Research Centre) | relapse mortality and survival at 200 days. The study does state that patients are required to stay within 30 minutes of the hospital for the first 80 to 100 days, which allows them to be closer (for most patients than their residential address) for any early issues. After this patients were followed up via telemedicine in addition to travelling to the clinics. |
| Meersman, et al. [109]<br><br>USA<br><br>2009 | Breast Cancer | California Health Interview survey<br><br>2001<br><br>Sample = 4,249 | Mammography uptake | Straight-line Distance.<br><br>Distances were treated as categorical variable and split into the following quartiles: 0 - 0.53 miles, 0.54 - 1.07 miles,1.09 - 1.82 miles and 1.83 - 26.5 miles. The study also calculated the number of public transit stops within 3 miles of the respondent and split these into quartiles. | Patients' Residence (70% of the sample were geocoded based on the nearest street to their residence, 30% to their zip code centroid).<br><br>TO<br><br>The nearest mammography facility. | The study did not use the distance calculations in the final model (as they were not significant)- but instead used mammography density within 2 miles of a patient's residence instead - which was found to be significant. The number of bus stops within 3 miles was not significant. This indicated that density of mammography facilities and not distance was the critical factor. |
| Ragon, et al. [110]<br><br>USA<br><br>2014 | Allogeneic hematopoietic stem cell transplantation (HSCT) | Transplant data team and medical records<br><br>2006 - 2012<br><br>Sample = 299 | Survival | Straight-line Distance.<br><br>Distance from the transplant centre was split into 2 groups of <170km and >170km. This represented a cut off at 75th percentile. | Patients Residence (Zip code at the time of the transplant)<br><br>TO<br><br>The medical centre where they were treated. | The study found that distance did not impact on the overall survival rate. |
| Sauerzapf, et al. [111]<br><br>UK<br><br>2008 | Breast Cancer | Northern and Yorkshire Cancer Registry Information Service.<br><br>1994 - 2002<br><br>Sample = 6,014 | Breast conserving surgery vs mastectomy & whether the patient had received radiotherapy following breast conserving surgery. | Travel Time.<br><br>Fastest Travel time using the road network. Using ArcGIS and the Meridian digital road network. Sections of the road were assigned average car travel times. Distances were treated as categorical variables using the categories of ≤30 mins, 30 - 60 mins > 60 mins. The study also collected information on those living within 800m of a frequent bus service. | Patients' Residence (postcode)<br><br>TO<br><br>The closest hospital where radiotherapy was available. | The study found that the choice of breast conserving surgery or receiving radiotherapy was not associated with the estimated travel time. They did find that travel time to radiotherapy was only significant as a predictor of surgery choice for patients living >800 m from a frequent bus service. |

| | | | | | | |
|---|---|---|---|---|---|---|
| Schroen and Lohr [112]<br><br>USA<br><br>2009 | Breast Cancer | Virginia Cancer Registry 2000 - 2001 Sample = 8,170 | Invasive tumour size at diagnosis | Shortest Road Distance.<br><br>Calculated using ArcGIS. Distance was treated as a continuous variable. The average distance was 5.7 miles and only 5% of the patients lived >20 miles away. | Patients' Residence<br><br>TO<br><br>The nearest mammography facility. | The study found that distance to the nearest mammography facility had no consistent relationship between invasive tumour size at diagnosis in the adjusted model. They found that only advanced age was a predictor of invasive tumour size at diagnosis |
| Other Studies | | | | | | |
| Firozvi, et al. [113]<br><br>USA<br><br>2008 | Liver Transplant | Medical Centre Transplant Database.<br><br>2002 - 2005 (censor date 2005)<br><br>Sample = 166. | Listing status, time required to list, survival once listed, transplantation and 1yr post transplantation survival. | Travel Time.<br><br>Calculated using Yahoo! Maps.<br><br>Travel time was treated as a categorical variable using > 3 hour and ≤3 hour. 38 people had travel times > 3. The range of travel times was 0 - 7 hours. | Patients' Residence (where not available the patients home town or city centre)<br><br>TO<br><br>The specific transplant centre | The study found that those patients living > 3 hours away from a transplant centre had comparable outcomes to those living closer. |
| Leese, et al. [114]<br><br>UK<br><br>2013 | Diabetes Related Foot Disease | Three linked data sets. Scottish Care Information Diabetes Collaboration - Tayside Regional Diabetes Register, Foot ulcer dataset, Amputation dataset.<br><br>2004 - 2006<br><br>Sample = 15,983. 670 (with new foot ulcers) 99 (with an amputation) | Occurrence of a new foot ulcer or amputation | Travel Time (using road distance)<br><br>Travel time was treated as a continuous variable. The average time to the GP was 6.48 minutes, average time to the local hospital was 28.47 minutes. | Patients' Residence<br><br>TO<br><br>The local hospital clinic and local GP | The study concluded that distance from the GP or hospital clinic and lack of attendance at community retinal screening did not predict a foot ulceration or amputation. They did find that being socially deprived was significantly associated with foot ulceration. |
| Markin, et al. [115]<br><br>USA<br><br>2011 | Pulmonary Arterial Hypertension | PAH Disease Management (REVEAL).<br><br>Years Unknown.<br><br>Sample = 638 | Delayed diagnosis | Distance.<br><br>(method not reported)<br>Distance was treated as a categorical variable using the grouping of < 50miles vs >50 miles. | Patients' Residence<br><br>TO<br><br>The pulmonary hypertension (PH) centre | The study concluded that distance from the PH centre was not shown to be associated with a delayed diagnosis, lower likelihood of early treatment with an IV/SC prostacyclin analog, or a worse functional class at diagnosis. |
| Rodkey, et al. [116]<br><br>USA | Heart Transplant | Transplantation hospital charts, local hospital records and direct patient and family | Rejection episodes, No. of endomyocar | Distance.<br><br>Distance was calculated using the Rand McNally TripMaker Version 1.1. | Primary city of residence<br><br>TO<br><br>The transplant centre | The study concluded that long distance management of heart transplant recipients is successful and is not associated with an increase in adverse outcomes. Patients living further away |

| | | | | | | |
|---|---|---|---|---|---|---|
| 1997 | | contact.<br>1984 - 1995<br>Sample = 312 | dial biopsies, ED visits, hospital admissions, infections, coronary allograft vasculopathy, malignancies re-transplantation and death | Distance was treated as a categorical variable using the groups 0 - 150miles 151 - 300 miles and >300miles. 207 patients lived in group 1, 69 patients lived in group 2 and 36 in group 3. (range 2 - 1218 miles) | | had similar results to those in the closest category (0 – 151 miles). |
| Stoller, et al. [117]<br>USA<br>2005 | 1-Antitrypsin (AAT) deficiency | The results are based on a 4 page mailed out survey to AAT deficient individuals. Achieving a 38% response rate.<br>2003<br>Sample = 1,851 (Achieving a 38% response rate) | Diagnostic delay | Distance.<br>Calculated using GIS software<br>Distance was treated as a categorical variable using the groups of < 50 miles and ≥ 50 miles to the CRC. 38% of the survey respondents lived within 50 miles of a CRC. | Patients' Residence ( zip code)<br>TO<br>The nearest designated clinical resource centre. | The study found that neither urban residence nor living near a centre with expertise (living within 50 miles) was associated with a shortened delay in diagnosis. |
| Swan-Kremeier, et al. [118]<br>USA<br>2005 | Eating Disorder | Contact records, clinical records and appointment records of patients at a treatment centre.<br>Unknown date.<br>Sample = 139 (37 completers & 102 drop outers) | Attendance Patterns and Treatment Attrition | Straight-line Distance.<br>Distance was treated as a continuous variable. The average distance for completers was 43.9 miles and the average distance for drop outers was 29.8 miles. | Patients' Residence<br>To<br>The treatment centre | The study concluded that distance travelled to the treatment site was not significantly different between the two groups (drop outers and completers). |
| Tonelli, et al. [119]<br>CANADA<br>2006 | Kidney transplantation | Canadian Organ Replacement Registry.<br>Patients starting dialysis 1996 - 2000 (followed until Dec 2001) | Likelihood of Transplant | Distance<br>(No information on distance calculations).<br>Distance was treated as a categorical variable using the groups - < 50km, 50.1 - 150km, 150.1 - 300km and > 300km. | Patients' Residence (at the time of starting dialysis)<br>TO<br>The nearest transplant centre | The study found that the likelihood of a transplant was not affected by the distance to the nearest transplant centre. |

| | | | | | | |
|---|---|---|---|---|---|---|
| | | Sample = 7,034 | | | | |
